# DeePVP: Identification and classification of phage virion proteins using deep learning

**DOI:** 10.1093/gigascience/giac076

**Published:** 2022-08-11

**Authors:** Zhencheng Fang, Tao Feng, Hongwei Zhou, Muxuan Chen

**Affiliations:** Microbiome Medicine Center, Department of Laboratory Medicine, Zhujiang Hospital, Southern Medical University, Guangzhou 510280, China; Microbiome Medicine Center, Department of Laboratory Medicine, Zhujiang Hospital, Southern Medical University, Guangzhou 510280, China; Microbiome Medicine Center, Department of Laboratory Medicine, Zhujiang Hospital, Southern Medical University, Guangzhou 510280, China; Microbiome Medicine Center, Department of Laboratory Medicine, Zhujiang Hospital, Southern Medical University, Guangzhou 510280, China

**Keywords:** phage virion protein, protein annotation, deep learning

## Abstract

**Background:**

Many biological properties of phages are determined by phage virion proteins (PVPs), and the poor annotation of PVPs is a bottleneck for many areas of viral research, such as viral phylogenetic analysis, viral host identification, and antibacterial drug design. Because of the high diversity of PVP sequences, the PVP annotation of a phage genome remains a particularly challenging bioinformatic task.

**Findings:**

Based on deep learning, we developed DeePVP. The main module of DeePVP aims to discriminate PVPs from non-PVPs within a phage genome, while the extended module of DeePVP can further classify predicted PVPs into the 10 major classes of PVPs. Compared with the present state-of-the-art tools, the main module of DeePVP performs better, with a 9.05% higher *F1-score* in the PVP identification task. Moreover, the overall *accuracy* of the extended module of DeePVP in the PVP classification task is approximately 3.72% higher than that of PhANNs. Two application cases show that the predictions of DeePVP are more reliable and can better reveal the compact PVP-enriched region than the current state-of-the-art tools. Particularly, in the *Escherichia* phage phiEC1 genome, a novel PVP-enriched region that is conserved in many other *Escherichia* phage genomes was identified, indicating that DeePVP will be a useful tool for the analysis of phage genomic structures.

**Conclusions:**

DeePVP outperforms state-of-the-art tools. The program is optimized in both a virtual machine with graphical user interface and a docker so that the tool can be easily run by noncomputer professionals. DeePVP is freely available at https://github.com/fangzcbio/DeePVP/.

## Introduction

Viruses are the dominant biological entities in the biosphere [1]. Because of the diversity of phage genomes, approximately 50–90% of phage genes cannot be assigned functions [2]. Additionally, it has been estimated that approximately 60–99% of viral metagenomic data do not have obvious homology to known sequences within databases [3]. Thus, a large number of viral genes exist as “dark matter,” which is a barrier to our understanding of viral genomes. Therefore, the development of gene function prediction tools for viral genomes is urgently needed.

Phage virion proteins (PVPs), also called phage structural proteins, are the proteins that make up viral particles, such as the head and tail. The comprehensive annotation of PVPs is essential for many phage genome analyses [4]. For example, marker genes (such as 16S ribosomal DNA in bacteria) are currently lacking for phages, but it has been suggested that some PVPs may be used as marker genes for phage genome analysis [5]. Additionally, analyses of PVPs in the phage genome could improve our understanding of phage–bacterial host interactions [6], direct antibacterial drug and antibiotic design [7], select specific phages for phage therapy [8], and assist in identifying prophages within bacterial genomes [9].

Historically, mass spectrometry has been the experimental method most commonly used for PVP identification [10]. With the rapid increase in viral sequencing data, low-cost and high-performing bioinformatic algorithms to perform PVP annotation are urgently needed. However, the diversity of PVPs is much higher than that of the enzymes encoded in the phage genome, which makes the identification of PVPs much more difficult [11]. To overcome this difficulty, several *de novo* algorithms for PVP identification have been proposed [8, 11–24]. The publication by Kabir et al. [25] provided a systematic review of most of these tools. Most of these tools are 2-class classifiers that can distinguish whether or not a given phage protein is a PVP. These tools were generally developed first by constructing a benchmark training and testing data set containing PVPs and non-PVPs from public databases and then using specific machine learning–based algorithms, such as support vector machine (SVM), to train and test the classifier using the data set. Among the tools mentioned above, PVPred [13], PVP-SVM [15], PVPred-SCM [20], Meta-iPVP [21], and VirionFinder [22] are available via a 1-click software package or a web server during the period of this work. In addition to these 2-class classification tools that distinguish PVPs and non-PVPs, other tools have been designed for identifying specific PVPs, such as the capsid and tail [5, 11]. Distinct from these tools, PhANNs [8] is a multiclass classifier that not only can identify whether a given protein is PVP but also further classifies the predicted PVP into one of several major PVP classes, making PhANNs a more powerful tool for PVP annotation.

Although these tools achieved better performance than the other tools available prior to their publication, further efforts can be made to improve PVP annotation. For example, most of these tools were trained and tested using small-scale data sets containing fewer than 1,000 proteins, which may not reflect the full diversity of PVP sequence features. With the rapid growth of public databases, more PVPs and non-PVPs can be included in the algorithm development processes. In terms of the protein sequence characterization method used, most of these tools used amino acid composition-based vectors; for example, PhANNs uses a *k*-mer vector. However, such vectors may be sparse and thus difficult to fit by the algorithm. Additionally, for many of the currently available tools mentioned above, a feature selection step must be performed before the feature vector is imported into the algorithm. Moreover, except for PhANNs, the current PVP annotation tools can only distinguish PVPs and non-PVPs or identify a certain class of PVPs; they cannot further classify PVPs into specific classes, which prevents detailed analysis of the phage genome.

To improve the performance of PVP annotation, we present DeePVP. DeePVP takes a phage protein as input; then, the main module of DeePVP outputs a PVP likelihood score to reflect whether the given protein belongs to the PVP, while the extended module further calculates whether a predicted PVP belongs to 1 of 10 major classes of PVPs, namely, head–tail joining, collar, tail sheath, tail fiber, portal, minor tail, major tail, baseplate, minor capsid, and major capsid, which are the dominant categories in PVP. Both modules use the one-hot encoding method to characterize the protein sequence and use a convolution neural network (CNN) as the classifier for protein feature extraction. Testing using the benchmark data set and 2 application cases demonstrate the advantages of DeePVP over existing tools in PVP annotation.

## Materials and Methods

Several benchmark training and testing data sets of PVP and non-PVP sequences have been constructed in previous work [8, 11–24]. In this work, the benchmark data set of PhANNs (http://edwards.sdsu.edu/phanns/download/expandedDB.tgz, downloaded on 17 September 2021) [8], which was constructed using proteins from the NCBI protein database and GenBank database, was used to develop DeePVP. This data set was chosen for the following reasons: (i) at the time of this work, the PhANNs data set is the largest PVP and non-PVP data set, containing a total of 168,660 PVPs and 369,553 non-PVPs, whereas most of the other data sets contain fewer than 1,000 proteins; (ii) the homology between the cross-validation and testing proteins is less than 40%, which is important in evaluating whether the algorithm can predict novel proteins, and within the cross-validation set, the sequence homology between the training and validation set in each rotation of the 10-fold cross validation is also less than 40%; and (iii) the data set includes all 10 major classes of PVPs that we focused on.

**Figure 1:**
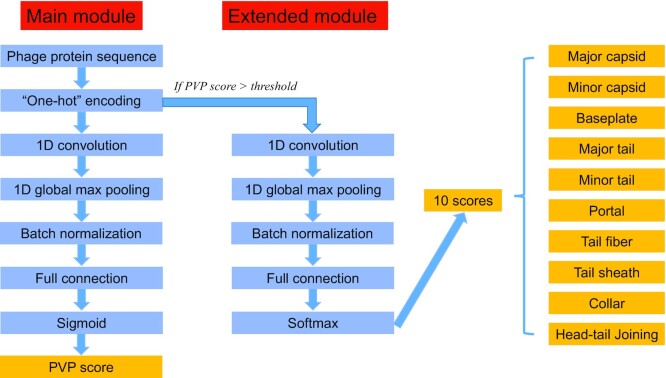
The framework of DeePVP. DeePVP takes a protein sequence as input, the main module calculates a PVP score representing the probability that the protein belongs to PVP, and the extended module calculates likelihood scores for each of the 10 PVP classes to determine the most likely class.

The framework of DeePVP is shown in Fig. 1. Selecting an appropriate representation method for biological sequences is an important step for bioinformatics algorithm development. Although the *k*-mer frequency vector has been widely used in many studies, such global statistics may lose certain local sequence information, such as information related to conserved domains or motifs in the sequence [26]. In DeePVP, we used the “one-hot” encoding form to represent the protein sequence. In this way, each amino acid is represented by a “one-hot” vector containing 20 bits, in which 19 bits are 0 and a certain bit is 1; therefore, the information of each amino acid is retained in the digitized model (see Section 1 of Additional File 1 for more details). Because of the data set size limitation, in addition to the sequence information, previous tools often included other hand-designed features to represent the sequence, such as some chemical properties of the corresponding amino acid. Considering that the data set for DeePVP contains a large number of protein sequences and that deep learning can extract meaningful features from large-scale raw data [27], we did not use additional information for the DeePVP construction.

It has been shown that CNN can effectively extract abstract features from “one-hot” protein sequence representations and make reliable classifications. For example, Google Research recently designed a tool named ProtCNN, which used the “one-hot” encoding form to represent a protein domain sequence and used a CNN to classify the domain into one member of the Pfam family [28]. In DeePVP, the “one-hot” encoded protein is first processed by the main module. The main module uses CNN to extract the sequence features to determine whether the given protein is a PVP. The CNN contains a 1-dimensional (1D) convolution layer, a 1D global max pooling layer, a batch normalization layer, a full connection layer, and, finally, a sigmoid layer that outputs a PVP score between 0 and 1. By default, a protein with a PVP score higher than 0.5 is regarded as a PVP. The extended module also uses CNN to classify the protein into a specific class of PVP. The CNN in the extended module contains a 1D convolution layer, a 1D global max pooling layer, a batch normalization layer, a full connection layer, and, finally, a softmax layer that outputs 10 likelihood scores representing the probability that the protein belongs to the head–tail joining, collar, tail sheath, tail fiber, portal, minor tail, major tail, baseplate, minor capsid, or major capsid class. By default, the category with the highest score will be chosen as the final prediction. The details of the hyperparameter selection are provided in Section 2 of Additional File 1.

In the training process, both the PVPs and non-PVPs in the training set were used to train the main module, and only PVPs were used to train the extended module. In the prediction process, each protein is processed through the main module and extended module. However, it is worth noting that if a protein obtains a PVP score lower than the threshold, the 10 scores calculated by the extended module will not make sense because this protein is not a PVP. Therefore, in the DeePVP workflow, we normalize the 10 scores from the extended module such that their sum is equivalent to the PVP score.

## Results

### Performance comparison in the PVP identification task

We first evaluated the PVP identification performance of the main module of DeePVP using 10-fold cross validation with the cross-validation set. The evaluation criteria are as follows:

(1) \begin{eqnarray*}
recall{\mathrm{\ }} = \frac{{TP}}{{TP + FN}}
\end{eqnarray*}

 

(2) \begin{eqnarray*}
precision{\mathrm{\ }} = \frac{{TP}}{{TP + FP}}
\end{eqnarray*}

 

(3) \begin{eqnarray*}
F1 - score{\mathrm{\ }} = {\mathrm{\ }}2 \times \frac{{recall \times precision}}{{recall + precision}}
\end{eqnarray*}

where *TP, TN, FN*, and *FP* represent the number of true-positive, true-negative, false-negative, and false-positive predictions, respectively. Among these 3 criteria, the *F1-score* can serve as a comprehensive index for evaluating PVP identification tools. We found that the main module of DeePVP achieved satisfactory performance, with average *recall, precision*, and *F1-score* values of 85.61%, 96.65%, and 90.76%, respectively.

We then trained the main module of DeePVP with all sequences in the cross-validation set and compared the performance between DeePVP and several state-of-the-art tools, namely, PVPred, PVP-SVM, PVPred-SCM, Meta-iPVP, PhANNs, and VirionFinder, using the test set. The performance comparison is shown in Table 1. Although the *recall* of DeePVP is slightly lower than that of PhANNs and VirionFinder, the *precision* of DeePVP is better than those of all the other tools, and the comprehensive index of *F1-score* is 9.05% higher than that of PhANNs, which performs the best among the other tools. Since DeePVP and PhANNs were trained using the same training data, the improved performance of DeePVP suggests that “one-hot” encoding may provide a more detailed representation method than the *k*-mer frequency used to characterize the protein sequences in PhANNs, and the deep convolution neural network in DeePVP may be more powerful than the simple shallow network containing 2 hidden layers used for feature extraction in PhANNs.

**Table 1:** Performance comparison of DeePVP and related tools in the PVP identification task

Tool	*Recall* (%)	*Precision* (%)	*F1-score* (%)
**DeePVP**	**88.10**	**96.75**	**92.22**
VirionFinder	90.91	44.00	59.30
PhANNs	91.68	76.11	83.17
Meta-iPVP	82.41	53.29	64.72
PVPred-SCM	41.28	38.71	39.95
PVP-SVM	41.31	46.19	43.62
PVPred	29.84	42.23	34.97

Selecting appropriate hyperparameters, such as the number of convolutional kernels and the kernel length, is one of the most important steps for constructing a robust and reliable neural network [27]. To test the advantage of the hyperparameters selected for DeePVP, we retrained DeePVP with different numbers of convolutional kernels and kernel lengths. As shown in Section 3 of Additional File 1, the performance achieved by DeePVP with other hyperparameters is not better than that of the CNN with the original hyperparameters, indicating that the hyperparameter design of DeePVP is suitable.

In the evaluation, we used 0.5 as the default threshold for DeePVP. In general, with a higher threshold, the *recall* will be lower, while the *precision* will be higher. In Section 4 of Additional File 1, we shown the *recall, precision*, and *F1-score* values yielded by DeePVP under different thresholds. In the released package of DeePVP, the output file also includes the PVP score for each protein, and the user can select a proper threshold according to their needs.

### Performance comparison in the PVP classification task

We further evaluated the PVP classification performance of the extended module of DeePVP. For each PVP category, we used the criteria of *recall, precision*, and *F1-score* to evaluate the performance of the tool, and we also used the *accuracy*, which was defined as the ratio of the correctly predicted sequences to the total number of sequences, to evaluate the overall performance of the tool. In the 10-fold cross-validation, the extended module of DeePVP again achieved satisfactory performance, with an average *accuracy* of 90.19%. The *recall, precision*, and *F1-score* for each PVP category in the cross-validation are shown in Section 5 of Additional File 1.

After training the extended module of DeePVP using all sequences in the cross-validation set, we further compared the performance between the extended module of DeePVP and PhANNs in the PVP classification task using the test set. Since the other tools cannot further classify a given PVP into a specific class, these tools were not included in this analysis. During the comparison, we assume that all the PVPs have already been correctly predicted in the upstream analysis; therefore, non-PVPs are excluded from the test set in this subsection. In practice, in the released package of DeePVP, the main module and the extended module can be run consecutively using an integrated pipeline or be run separately. For example, if researchers have already identified PVPs using other computational or experimental methods, such as mass spectrometry, they can run the extended module of DeePVP directly for PVP classification.

We used the criteria of *recall, precision*, and *F1-score* to evaluate the performance of DeePVP and PhANNs for each PVP category. We also used *accuracy* to evaluate the overall performance of the tools. The performance comparison is shown in the Table 2. The overall *accuracy* of DeePVP is 3.72% higher than that of PhANNs, indicating that DeePVP has a better ability to classify PVPs. In terms of each category, although the *F1-score* values of DeePVP for the major tail, tail fiber, tail sheath, and collar are lower than those of PhANNs, DeePVP presents a higher *F1-score* for other categories. While the *accuracy* advantage of DeePVP is not as prominent, as it is only 3.72% higher than that of PhANNs, it is worth noting that such performance is evaluated under the assumption that all PVPs have been correctly predicted. Since DeePVP performs better than PhANNs in PVP identification, we consider that the comprehensive PVP annotation performance of DeePVP is better than that of PhANNs.

**Table 2:** Performance comparison of DeePVP and PhANNs in the PVP classification task

Category	Tool	*Recall* (%)	*Precision* (%)	*F1-score* (%)	*Accuracy* (%)
Major capsid	DeePVP	98.58	90.88	94.57	NA
	PhANNs	91.98	94.24	93.10	
Minor capsid	DeePVP	50.62	44.09	47.13	
	PhANNs	80.25	13.98	23.81	
Baseplate	DeePVP	86.49	95.96	90.98	
	PhANNs	78.85	88.76	83.51	
Major tail	DeePVP	77.09	61.23	68.25	
	PhANNs	80.48	86.88	83.56	
Minor tail	DeePVP	90.28	93.24	91.74	
	PhANNs	85.42	90.50	87.89	
Portal	DeePVP	93.10	93.62	93.36	
	PhANNs	85.88	93.77	89.65	
Tail fiber	DeePVP	78.24	68.42	73.00	
	PhANNs	77.93	69.66	73.56	
Tail sheath	DeePVP	92.86	99.42	96.03	
	PhANNs	94.39	99.64	96.94	
Collar	DeePVP	36.33	80.15	50.00	
	PhANNs	87.00	75.65	80.93	
Head–tail joining	DeePVP	96.48	96.33	96.40	
	PhANNs	88.49	71.88	79.33	
**All**	**DeePVP**		NA		**91.06**
	PhANNs				87.34

NA: not applicable.

### Application case 1: PVP annotation of the mycobacteriophage PDRPxv genome

To demonstrate the value and reliability of DeePVP in PVP annotation, we first used DeePVP and related tools to perform PVP annotation of the genome of the mycobacteriophage PDRPxv (GenBank accession: KR029087, downloaded on 30 September 2021), which is considered a candidate therapeutic for pathogenic *Mycobacterium* species [29]. The PVPs in the PDRPxv genome have been identified experimentally by mass spectrometry; such experimental data can be used to evaluate the reliability of computational tools. The tools DeePVP, PVPred, PVP-SVM, PVPred-SCM, Meta-iPVP, PhANNs, and VirionFinder were used to perform PVP annotation of the PDRPxv genome, and the mass spectrometry data were used to evaluate the *recall, precision*, and *F1-score* of each tool. The performance of each tool in the PVP identification task is shown in Table 3. We found that most of the compared tools did not perform well, with *F1-scores* lower than 50%. PhANNs performed better than the other tools, and the *F1-score* of DeePVP was 21.19% higher than that of PhANNs, indicating that DeePVP provides a more reliable prediction.

**Table 3:** Performance comparison in the PVP identification task in the PDRPxv genome, validated according to mass spectrometry data

Tool	*Recall* (%)	*Precision* (%)	*F1-score* (%)
**DeePVP**	**75.00**	**100.00**	**85.71**
VirionFinder	91.67	27.50	42.31
PhANNs	83.33	52.63	64.52
Meta-iPVP	75.00	15.79	26.09
PVPred-SCM	58.33	12.28	20.29
PVP-SVM	58.33	17.07	26.42
PVPred	50.00	18.18	26.67

**Figure 2:**
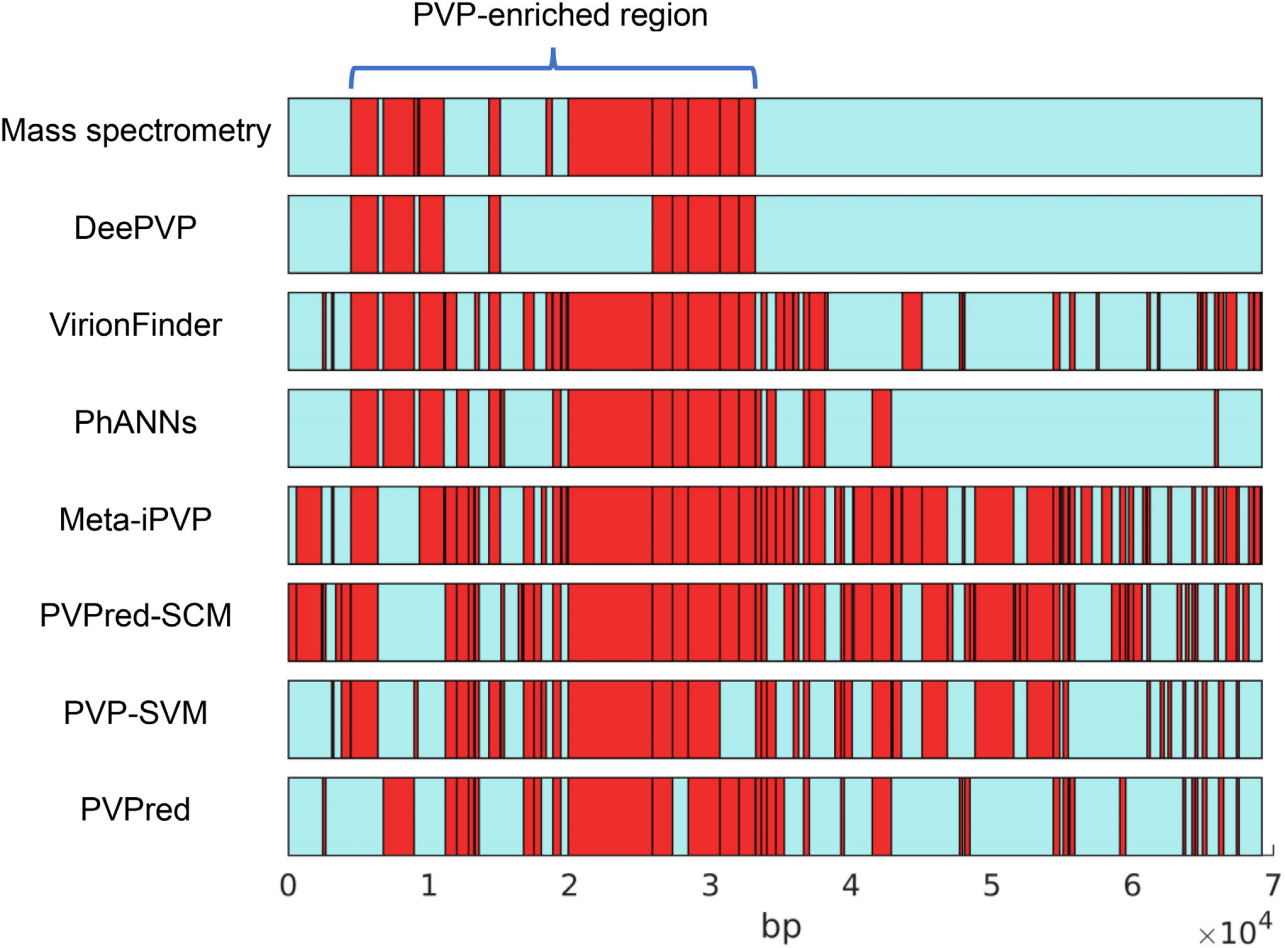
The distribution of the PVPs uncovered by mass spectrometry and the PVPs predicted by each tool on the mycobacteriophage PDRPxv genome. The blue bars represent the PDRPxv genome, and the red boxes represent the PVPs. The horizontal axis represents the base coordinates.

In Fig. 2, we show the base coordinates for the PVPs uncovered by mass spectrometry and the PVPs predicted by each tool. The mass spectrometry data showed that all PVPs were located within a compact PVP-enriched region, and no PVPs were identified outside the PVP-enriched region. In fact, it has been shown that PVPs are often located near each other within the genome [30,31], and such PVP distribution patterns may be common among phages. Interestingly, we found that the PVP distribution pattern revealed by DeePVP was quite consistent with that revealed by mass spectrometry. Although DeePVP failed to predict a few PVPs, all of the other predicted PVPs were located within the PVP-enriched region. In contrast, the PVPs predicted by the other tools seem to be distributed randomly throughout the genome. This phenomenon shows that, in comparison with other tools, DeePVP provides more reliable prediction and has a better ability to reveal the genomic structure of a phage genome.

According to mass spectrometry, the PDRPxv genome contains 12 PVPs. Sinha et al. [29] inferred the putative function of each PVP through a combination of several strategies, including domain search, homology analysis, adjacent gene analysis, and protein secondary structure analysis. In Table 4, we compared the PVP classes predicted by DeePVP and PhANNs with the putative functions revealed by Sinha et al. We found that the predictions of DeePVP and PhANNs were basically consistent with the putative functions. In particular, 5 putative minor tail proteins are encoded continuously (Gp29–Gp33) in the genome, and both DeePVP and PhANNs predicted this minor tail protein cluster appropriately.

**Table 4:** PDRPxv genome PVP classification by DeePVP and PhANNs

Protein ID	Putative function^a^	DeePVP prediction	PhANNs prediction
Gp8	Portal protein	Portal	Portal
Gp10	Minor head protein	Portal	Portal
Gp11	Scaffolding protein	NA^b^	NA
Gp12	Major capsid protein	Major capsid	Major capsid
Gp18	Major tail subunit	Major tail	Major tail
Gp25	Tail assembly chaperone	NA	NA
Gp28	Tape measure protein	NA	Minor tail
Gp29	Minor tail protein	Minor tail	Minor tail
Gp30	Minor tail protein	Minor tail	Minor tail
Gp31	Minor tail protein	Minor tail	Minor tail
Gp32	Minor tail protein	Minor tail	Minor tail
Gp33	Minor tail protein	Minor tail	Minor tail

a Putative function as analysed by Sinha et al. [29].

b NA indicates that the tool did not identify the corresponding protein as a PVP.

### Application case 2: A novel conserved PVP-enriched region was found in *Escherichia* phage phiEC1

**Figure 3:**
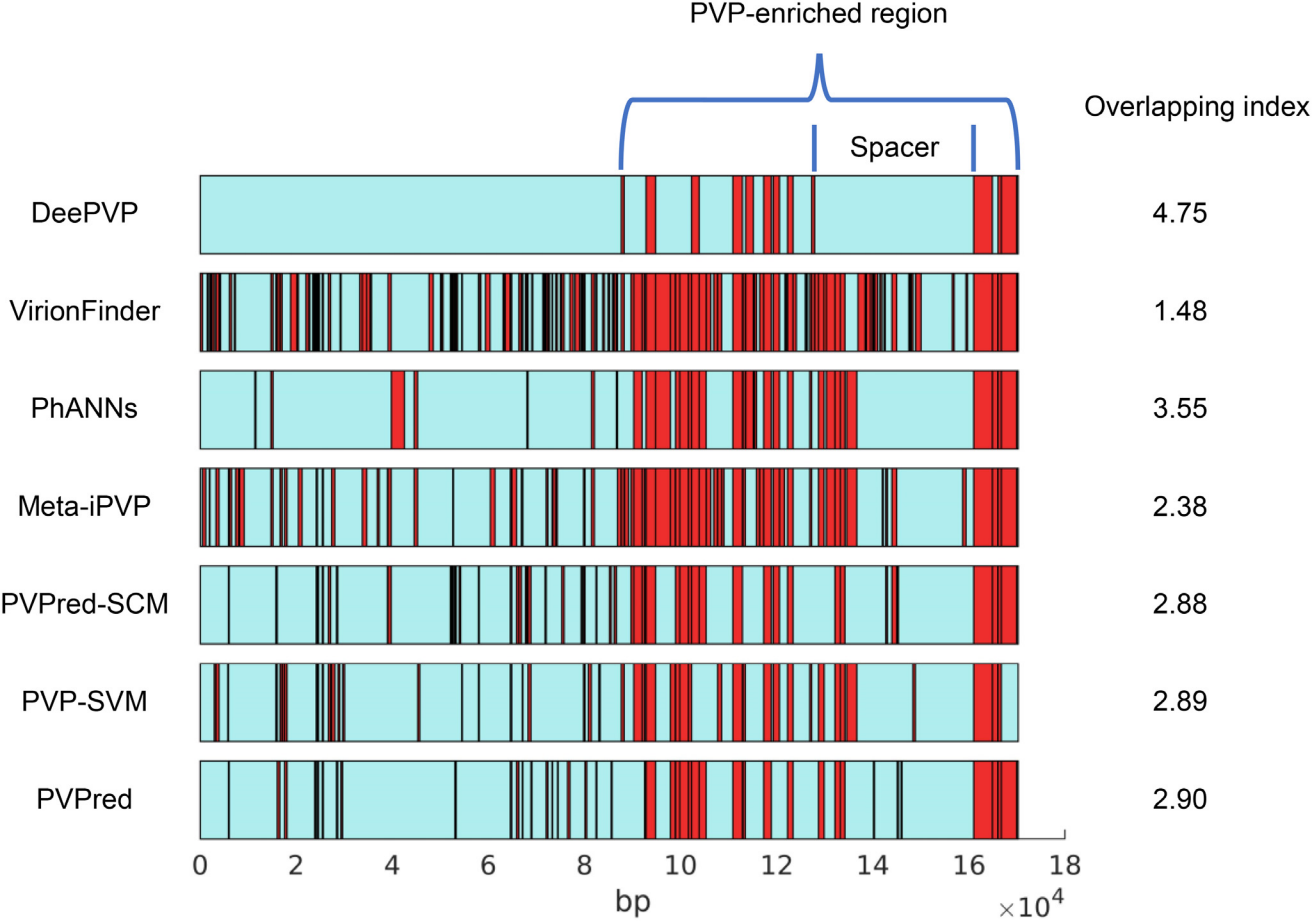
The distribution of PVPs predicted by each tool in the *Escherichia* phage phiEC1 genome. The blue bars represent the *Escherichia* phage phiEC1 genome, and the red boxes represent the predicted PVPs. The average overlapping index of each tool is shown on the right side of the bar. The genomic features revealed by DeePVP are also marked in the first bar. The horizontal axis represents the base coordinates.

Viral genomes are highly diverse, which presents a challenge to understanding viral evolutionary mechanisms [32]. Phage diversity is sometimes driven by PVPs [11]. In this subsection, we used DeePVP to perform PVP annotation on *Escherichia* phage phiEC1 (RefSeq accession: NC_041920.1, downloaded on 20 June 2021), a phage that may be an effective treatment for murine models of bacteremia [33], to reveal its genomic features. According to the annotation of the RefSeq database, the *Escherichia* phage phiEC1 genome encodes 269 proteins, all of which lack functional annotation information in the RefSeq database. Additionally, no experimental data are available to determine which proteins are PVPs; therefore, PVP annotation using computational methods is an efficient way to analyze the genome. We used all the related tools to perform PVP annotation on the phage phiEC1 genome. The base coordinates for the PVPs predicted by each tool are shown in Fig. 3. We defined a “PVP prediction overlapping index” for each PVP predicted by each tool to speculate the prediction reliability. For a certain protein predicted as a PVP by a certain tool, if this protein was also predicted as a PVP by *n* tools simultaneously, then the “overlapping index” for this predicted PVP was *n*. The average “overlapping index” of each tool is shown in Fig. 3. We found that DeePVP achieved the highest index of 4.75, indicating that PVP predicted by DeePVP is more often predicted by other tools.

Additionally, we found that the genomic features of the phage phiEC1 genome revealed by DeePVP were interesting. Compared with the PVP distributions of other tools, which were disperse across the genome, the distribution of all PVPs predicted by DeePVP was highly compact; a PVP-enriched region was clearly present, and there were no predicted PVPs outside the PVP-enriched region. This phenomenon is quite consistent with that of mycobacteriophage PDRPxv in application case 1, in which all PVPs are compactly encoded on the genome. Since such compact PVP distribution patterns are common [30,31], we consider that DeePVP may perform better in revealing the features of the phage genome. Meanwhile, from the DeePVP prediction results, we found that there was a clear spacer without predicted PVPs within the PVP-enriched region, as shown in Fig. 3.

**Figure 4:**
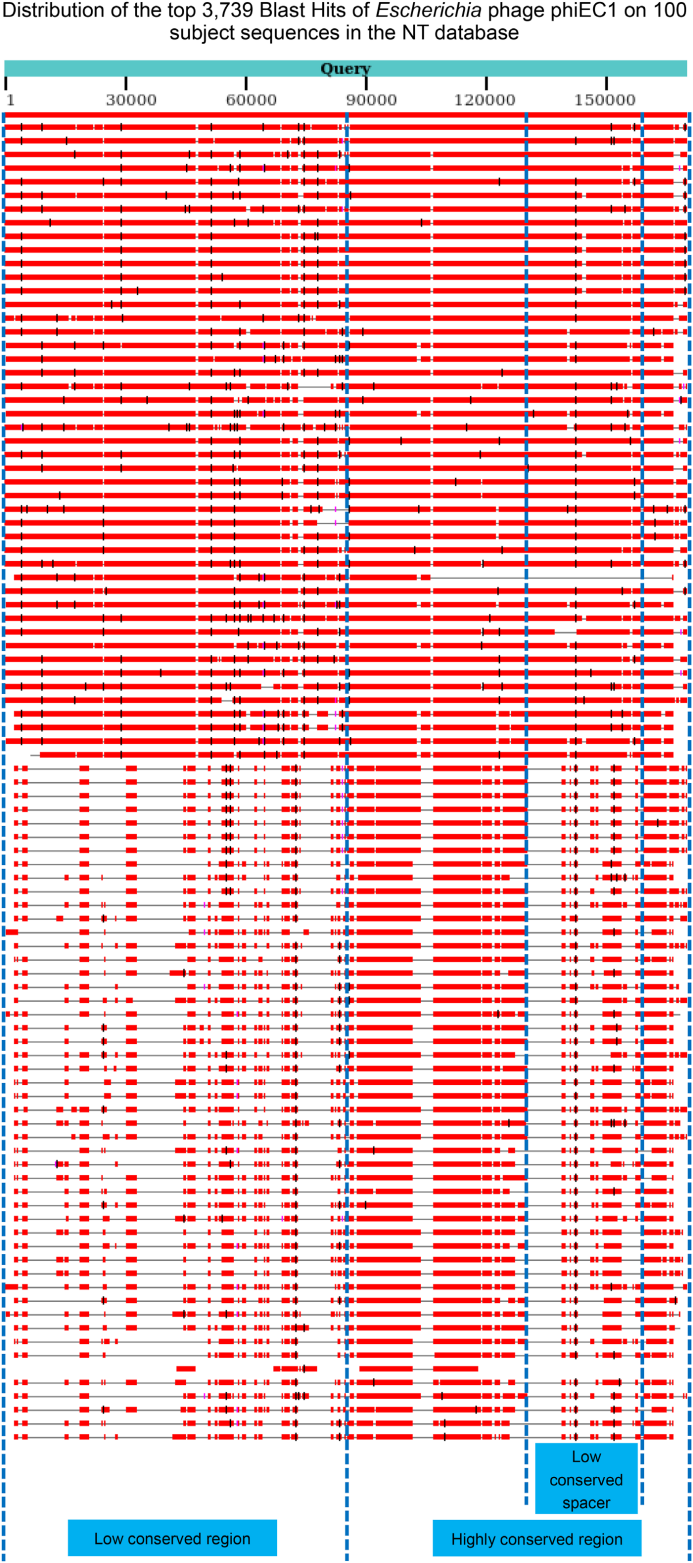
The alignment results of the top 100 subject sequences for the *Escherichia* phage phiEC1 genome. The blue bar represents the phage phiEC1 genome query sequence. Each line below the query sequence represents a certain subject sequence, and the colored box on the subject sequence represents a certain blast hit.

To perform further comparative genomic analysis, we aligned the phage phiEC1 genome to the NT database using the NCBI blastn online server (https://blast.ncbi.nlm.nih.gov/Blast.cgi), with all parameters at the default settings. The alignment results of the top 100 subject sequences are shown in Fig. 4, and detailed information about each subject sequence obtained from the server is provided in Additional File 2. All subject sequences were phage sequences. The alignment graph showed that the phage phiEC1 genome contained a low conserved region, which was conserved in some phage genomes but lacked obvious homology in other genomes, and a highly conserved region, which was present in almost all subject sequences. In addition, within the highly conserved region, there was a low conserved spacer that was less conserved among the subject sequences. More interestingly, when comparing the DeePVP prediction in Fig. 3 with the alignment results in Fig. 4, we found that the highly conserved region corresponded to the PVP-enriched region, while the low conserved region corresponded to the region outside of the PVP-enriched region. Additionally, the low conserved spacer within the highly conserved region approximately corresponded to the spacer within the PVP-enriched region. The above phenomenon suggests that the *Escherichia* phage phiEC1 genome contains a compact PVP-enriched region that has been conserved during viral evolution and that this PVP-enriched region contains a non-PVP spacer that might have been generated through recombination or horizontal gene transfer.

**Figure 5:**
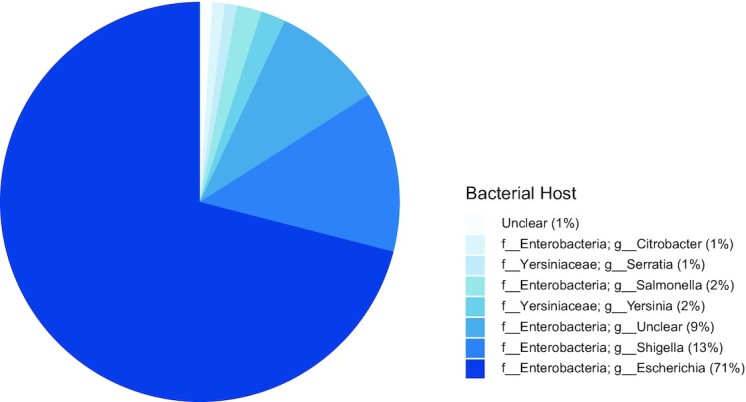
Host compositions of the subject sequences. We calculated the frequency of each host family and genus among the alignment results of *Escherichia* phage phiEC1 and found that most of the subject sequences were also *Escherichia* phages. The frequency of each host family and genus is shown in parentheses.

Moreover, the alignment results showed that most of the subject sequences (71% in total, including the phage phiEC1 genome itself) were also *Escherichia* phages, as shown in Fig. 5. Such results indicate that among phages that infect the same host, the PVP-enriched region may be more conserved than the non-PVP region during the viral evolution process. Generally, the host of a phage is determined by the interactions between the PVPs and the host cell receptors [34]; recently, a tool for phage host prediction based on PVP sequences was developed [6]. It is thus expected that DeePVP may be employed for the related processes of the phage–host prediction in the future.

The PVP classification results of DeePVP and PhANNs are shown in Table 5 and Section 6 of Additional File 1, respectively. We found that the categories predicted by DeePVP covered most of the essential PVPs for the viral particle, including the major capsid, major tail, tail sheath, tail fiber, baseplate, and portal. Additionally, 3 tail-associated PVPs, tail fibers, are encoded next to each other (YP_009965877.1, YP_009965879.1, YP_009965880.1). This phenomenon is similar to that of application case 1, in which 5 putative tail-associated PVPs, minor tails, are encoded continuously in the genome.

**Table 5:** PVP category predicted by DeePVP on the *Escherichia* phage phiEC1 genome

Protein ID	DeePVP prediction
YP_009965784.1	Tail sheath
YP_009965791.1	Baseplate
YP_009965797.1	Tail fiber
YP_009965805.1	Tail sheath
YP_009965807.1	Portal
YP_009965812.1	Major capsid
YP_009965814.1	Major capsid
YP_009965818.1	Major tail
YP_009965826.1	Baseplate
YP_009965877.1	Tail fiber
YP_009965879.1	Tail fiber
YP_009965880.1	Tail fiber

## Discussion

To develop DeePVP, we chose to train a 2-class classifier and a 10-class classifier separately rather than to train an 11-class classifier directly. This is because the number of protein sequences in each category of the data set is unbalanced. For example, in the data set, 369,553 of the proteins belong to the non-PVP category, while only 2,105 proteins belong to the collar category. An unbalanced data set presents a challenge for training a neural network because the neural network may tend to assign most of the samples to the category with the largest size automatically. In DeePVP, we first trained a CNN to separate PVP and non-PVP sequences; this reduces the impact of the non-PVP category, which contains many more sequences than the other PVP categories, on the PVP classification task.

Among the related tools, amino acid composition-based feature vectors are commonly used to characterize sequences. Such global statistics may fail to capture certain local information, such as conserved domains or motifs, in the sequence. Additionally, such feature vectors may be sparse. For example, PhANNs uses a *k*-mer-based feature vector containing thousands of bits, while a large number of proteins contain only approximately 250 amino acids (aa); therefore, most of the bits in the feature vector are likely to be 0. Such sparse vectors may make it difficult for the algorithm to fit the data. In DeePVP, we used “one-hot” encoding for the sequence, such that the information about each amino acid was retained in the characterization model. We then used CNN to extract the useful features from the raw data. It has been shown that CNN is powerful for extracting useful features, and the convolution kernel may serve as a sensitive position weight matrix to detect local specific motifs [35–37]. We therefore consider that this method may help to improve the performance of DeePVP.

Although the benchmark data set we used in this work is by far the largest and best-designed PVP and non-PVP data set currently available, it is primarily focused on the 10 major classes of PVPs and was created using keyword searches of public databases. Thus, some less frequently occurring PVPs may be excluded from the data set. In application case 1, we found that DeePVP failed to identify a putative scaffolding protein (Gp11) and a putative tail assembly chaperone (Gp25), which may be because these 2 types of proteins were not included in the data set by the keyword search process. Additionally, it is important to know how DeePVP judges PVPs that do not belong to these 10 types. We downloaded 340 tail fiber assembly proteins that were explicitly excluded from the PhANNs data set [8]. We found that 147 of them were predicted as PVP by the main module, and among these 147 predicted PVPs, 146 of them were predicted as tail-associated PVP classes by the extended module, including major tail, minor tail, tail fiber, and head–tail joining. This result shows that for a PVP that does not belong to the 10 types, the extended module of DeePVP may classify this PVP into the most relevant category. In the future, more efforts should be made to create more exhaustive PVP sets to further improve the algorithm performance. Additionally, the numbers of instances of some PVP types in the PhANNs data set are low, which may lead to poor performance for these types. For example, the minor capsid category contains the least number of sequences, with only 1,055 sequences included, and both PhANNs and DeePVP achieve low *F1-scores* on this class. Therefore, to improve the performance on PVPs with small data sizes, more sequences should also be added to such categories in the future with the continuous expansion of the public database.

Although the comprehensive index *F1-score* of the main module of DeePVP is better than that of the other tools, the *recall* of DeePVP is slightly lower than that of some tools, such as PhANNs and VirionFinder, in the PVP identification task, indicating that DeePVP may fail to identify some PVPs. On the other hand, DeePVP was better able to identify the PVP-enriched region, and PVPs are encoded compactly within a single region in many phages. Therefore, we suggest that users can combine DeePVP with other tools to identify as many PVPs as possible with a low false-positive prediction rate. In application case 1, for example, the user can first use DeePVP to identify the PVP-enriched region (from Gp8 to Gp33, as shown in Fig. 2 and Table 4). Then, within this region, users can apply the more sensitive tool PhANNs to identify more PVPs. Since proteins within the region are more likely to be PVPs, such an operation may be less likely to generate false-positive predictions. With this approach, the PVP of Gp28, which fails to be predicted by DeePVP, was included in the prediction, and the *recall* was increased from 75% to 83.33%. Although this approach also introduced 3 false-positive predictions (Gp14, Gp19, Gp26), a large number of false-positive predictions outside the PVP-enriched region are excluded. In the future, it would be worth constructing a comprehensive workflow that integrates different algorithms to improve the PVP annotation performance.

In metagenomic data, because of the difficulty of sequence assembly, some proteins exist as partial genes. Although the current version of DeePVP is primarily designed for complete phage genomes, we also test the performance of DeePVP on partial genes. The results in Section 7 of Additional File 1 show that DeePVP still achieves relatively satisfactory performance when the partial gene is longer than 50% of the full length, indicating that DeePVP is still capable of working with long contigs in metagenomic data.

## Conclusion

In this work, we present DeePVP, a new tool for PVP annotation of a phage genome. The main module of DeePVP aims to identify whether a phage protein is a PVP, while the extended module can further judge the class to which the PVP belongs. Evaluation using the large-scale benchmark data set shows that DeePVP performs better than the related tools. We provided 2 application cases to demonstrate the value of DeePVP. In the case of mycobacteriophage PDRPxv, by referring to experimental mass spectrometry data, we illustrated that the prediction of DeePVP was more reliable and could better reveal the compact distribution pattern of PVP over the genome than those of other tools. We then used DeePVP to perform PVP prediction on *Escherichia* phage phiEC1, which previously lacked experimental data and annotation information for its PVPs. Compared with the other tools, DeePVP again showed a clear PVP-enriched region within the genome, and we found that this newly discovered PVP-enriched region is conserved in many other phages that infect the same host genus during the viral evolution process. We therefore suggest that DeePVP may be a powerful tool for various phage genomic analysis applications, such as host prediction. DeePVP software is optimized in both a virtual machine with graphical user interface and a docker, which makes the software easy to install on a local PC or high-performance computing system. Noncomputer professionals who are not familiar with the command line can also easily run the tool on large-scale data.

## Availability of Supporting Source Code and Requirements

Project name: DeePVP.

Project home page: https://github.com/fangzcbio/DeePVP/.

Operating system: The code of DeePVP was written on Linux. We optimized the program in a virtual machine and docker thus DeePVP is platform independent.

Programming language: python, matlab.

Other requirements: no other requirements are needed.

License: GPL-3.0.

RRID: SCR_022474.

biotoolsID: DeePVP.

## Availability of Supporting Data

The supporting data related to the manuscript are available in the *GigaScience* repository, GigaDB [38].

## Additional Files

**Additional File 1**. Section 1: description of the one-hot encoding form; Section 2: description of the details of the hyperparameter selection of the neural network; Section 3: the performance achieved by DeePVP with different numbers of convolutional kernels and different kernel lengths on the PVP identification task; Section 4: the *recall, precision*, and *F1-score* values produced by DeePVP under different thresholds in the PVP identification task; Section 5: the average of *recall, precision, F1-score*, and *accuracy* of the extended module of DeePVP in the 10-fold cross-validation; Section 6: PVP category predicted by PhANNs on the *Escherichia* phage phiEC1 genome; Section 7: DeePVP performance on partial genes.

**Additional File 2**. Detailed information about each subject sequence of *Escherichia* phage phiEC1 genome obtained from the NCBI blastn server.

giac076_GIGA-D-22-00026_Original_Submission

giac076_GIGA-D-22-00026_Revision_1

giac076_GIGA-D-22-00026_Revision_2

giac076_GIGA-D-22-00026_Revision_3

giac076_Response_to_Reviewer_Comments_Original_Submission

giac076_Response_to_Reviewer_Comments_Revision_1

giac076_Response_to_Reviewer_Comments_Revision_2

giac076_Reviewer_1_Report_Original_Submission Satoshi Hiraoka -- 3/9/2022 Reviewed

giac076_Reviewer_1_Report_Revision_1 Satoshi Hiraoka -- 6/17/2022 Reviewed

giac076_Reviewer_2_Report_Original_Submission Deyvid Amgarten -- 3/19/2022 Reviewed

giac076_Reviewer_2_Report_Revision_1 Deyvid Amgarten -- 6/16/2022 Reviewed

giac076_Supplemental_Files

## Abbreviations

CNN: convolution neural network; PVP: phage virion protein; SVM: support vector machine.

## Competing Interests

The authors declare that they have no competing interests.

## Funding

This investigation was financially supported by the National Natural Science Foundation of China (82102508, 81925026, 82002201, 81800746).

## Authors' Contributions

ZCF and HWZ proposed and designed the study. ZCF and TF constructed the data sets and wrote and optimized the code. ZCF and MXC wrote and revised the manuscript and all authors proofread and improved the manuscript.

## Notes

Institutional addresses: 253 Gongye Middle Avenue, Haizhu District, Guangzhou, Guangdong, China, 510280.
