# Supplementary material for: DeePVP: Identification and classification of phage virion proteins using deep learning
Source: Gigascience. 2022 Aug 11;11:giac076. doi: 10.1093/gigascience/giac076 (PMC9366990; doi:10.1093/gigascience/giac076)
Supplement: giac076_GIGA-D-22-00026_Original_Submission [file giac076_giga-d-22-00026_original_submission.pdf]

GigaScience

DeePVP: Identification and classification of phage virion proteins using deep learning

--Manuscript Draft--

**Manuscript Number:**	GIGA-D-22-00026
**Full Title:**	DeePVP: Identification and classification of phage virion proteins using deep learning
**Article Type:**	Technical Note
**Funding Information:**	National Natural Science Foundation of China (82102508) — Dr. Zhencheng Fang<br>National Natural Science Foundation of China (81925026) — Prof. Hongwei Zhou<br>National Natural Science Foundation of China (82002201) — Dr. Muxuan Chen<br>National Natural Science Foundation of China (81800746) — Not applicable
**Abstract:**	Background: Many biological properties of phages are determined by phage virion proteins (PVPs), and the poor annotation of PVPs is a bottleneck for many areas of viral research, such as viral phylogenetic analysis, viral host identification and antibacterial drug design. Because of the high diversity of PVP sequences, the PVP annotation of a phage genome remains a particularly challenging bioinformatic task.<br>Findings: Based on deep learning, we developed DeePVP. The main module of DeePVP aims to discriminate PVPs from non-PVPs within a phage genome, while the extended module of DeePVP can further classify predicted PVPs into the ten major classes of PVPs. Compared with the present state-of-the-art tools, the main module of DeePVP performs much better, with a 9.05% higher F1-score in the PVP identification task. Moreover, the overall accuracy of the extended module of DeePVP in the PVP classification task is approximately 3.72% higher than that of PhANNs. Two application cases show that the predictions of DeePVP are much more reliable and can better reveal the compact PVP-enriched region than the current state-of-the-art tools. Particularly, in the Escherichia phage HP3 genome, a novel PVP-enriched region that is conserved in many other Escherichia phage genomes was identified, indicating that DeePVP will be a useful tool for phage host prediction by revealing PVP biomarkers that have been conserved during viral evolution.<br>Conclusions: DeePVP outperforms state-of-the-art tools. The program is optimized in both a virtual machine with GUI and a docker so that the tool can be easily run by non-computer professionals.
**Corresponding Author:**	Hongwei Zhou<br>Zhujiang Hospital<br>Guangzhou, CHINA
**Corresponding Author Secondary Information:**	
**Corresponding Author's Institution:**	Zhujiang Hospital
**Corresponding Author's Secondary Institution:**	
**First Author:**	Zhencheng Fang
**First Author Secondary Information:**	
**Order of Authors:**	Zhencheng Fang
	Tao Feng
	Hongwei Zhou
	Muxuan Chen

Powered by Editorial Manager® and ProduXion Manager® from Aries Systems Corporation

Order of Authors Secondary Information:	
Additional Information:	
Question	Response
Are you submitting this manuscript to a special series or article collection?	No
**Experimental design and statistics**<br><br>Full details of the experimental design and statistical methods used should be given in the Methods section, as detailed in our Minimum Standards Reporting Checklist. Information essential to interpreting the data presented should be made available in the figure legends.<br><br>Have you included all the information requested in your manuscript?	Yes
**Resources**<br><br>A description of all resources used, including antibodies, cell lines, animals and software tools, with enough information to allow them to be uniquely identified, should be included in the Methods section. Authors are strongly encouraged to cite Research Resource Identifiers (RRIDs) for antibodies, model organisms and tools, where possible.<br><br>Have you included the information requested as detailed in our Minimum Standards Reporting Checklist?	Yes
**Availability of data and materials**<br><br>All datasets and code on which the conclusions of the paper rely must be either included in your submission or deposited in publicly available repositories (where available and ethically appropriate), referencing such data using a unique identifier in the references and in the "Availability of Data and Materials" section of your manuscript.	Yes

Have you have met the above requirement as detailed in our Minimum Standards Reporting Checklist?	

Manuscript Click here to access/download;Manuscript;Manuscript.pdf

DeePVP: Identification and classification of phage virion proteins using deep learning

Zhencheng Fang[1†], Tao Feng[1†], Hongwei Zhou[1*] and Muxuan Chen[1*]

†These authors contributed equally to this work.

[1]Microbiome Medicine Center, Department of Laboratory Medicine, Zhujiang Hospital, Southern Medical University, Guangzhou, China.

*To whom correspondence should be addressed.

Email: hzhou@smu.edu.cn, chenmuxuan@126.com.

Institutional addresses:

253 Gongye Middle Avenue, Haizhu District, Guangzhou, Guangdong, China, 510280

Abstract

Background: Many biological properties of phages are determined by phage virion proteins (PVPs), and the poor annotation of PVPs is a bottleneck for many areas of viral research, such as viral phylogenetic analysis, viral host identification and antibacterial drug design. Because of the high diversity of PVP sequences, the PVP annotation of a phage genome remains a particularly challenging bioinformatic task.

Findings: Based on deep learning, we developed DeePVP. The main module of DeePVP aims to discriminate PVPs from non-PVPs within a phage genome, while the extended module of DeePVP can further classify predicted PVPs into the ten major classes of PVPs. Compared with the present state-of-the-art tools, the main module of DeePVP performs much better, with a 9.05% higher *F1-score* in the PVP identification task. Moreover, the overall *accuracy* of the extended module of DeePVP in the PVP classification task is approximately 3.72% higher than that of PhANNs. Two application cases show that the predictions of DeePVP are much more reliable and can better reveal the compact PVP-enriched region than the current state-of-the-art tools. Particularly, in the *Escherichia* phage HP3 genome, a novel PVP-enriched region that is conserved in many other *Escherichia* phage genomes was identified, indicating that DeePVP will be a useful tool for phage host prediction by revealing PVP biomarkers that have been conserved during viral evolution.

Conclusions: DeePVP outperforms state-of-the-art tools. The program is optimized in both a virtual machine with GUI and a docker so that the tool can be easily run by non-computer professionals.

Keywords

phage virion protein, protein annotation, deep learning

Introduction

Viruses are the dominant biological entities in the biosphere [1]. Because of the diversity of phage genomes, approximately 50-90% of phage genes cannot be assigned functions [2]. Additionally, it has been estimated that approximately 60-99% of viral metagenomic data do not have obvious homology to

known sequences within databases [3]. Thus, a large number of viral genes exist as “dark matter”, which is a barrier to our understanding of viral genomes. Therefore, the development of gene function prediction tools for viral genomes is urgently needed.

Phage virion proteins (PVPs), also called phage structural proteins, are the proteins that make up viral particles, such as the head and tail. The comprehensive annotation of PVPs is essential for many phage genome analyses [4]. For example, marker genes (such as 16S rDNA in bacteria) are currently lacking for phages, but it has been suggested that some PVPs may be used as marker genes for phage genome analysis [5]. Additionally, analyses of PVPs in the phage genome could improve our understanding of phage-bacterial host interactions [6], direct antibacterial drug and antibiotic design [7], select specific phages for phage therapy [8] and assist in identifying prophages within bacterial genomes [9].

Historically, mass spectrometry has been the experimental method most commonly used for PVP identification [10]. With the rapid increase in viral sequencing data, low-cost and high-performing bioinformatic algorithms to perform PVP annotation are urgently needed. However, the diversity of PVPs is much higher than that of the enzymes encoded in the phage genome, which makes the identification of PVPs much more difficult [11]. To overcome this difficulty, several de novo algorithms for PVP identification have been proposed [8,11-23]. Most of these tools are two-class classifiers that can distinguish whether or not a given phage protein is a PVP. These tools were generally developed first by constructing a benchmark training and testing dataset containing PVPs and non-PVPs from public databases and then using specific machine learning-based algorithms, such as support vector machine (SVM), to train and test the classifier using the dataset. Among the tools mentioned above, PVPred [13], PVP-SVM [15], PVPred-SCM [20], Meta-iPVP [21] and VirionFinder [22] are available via a one-click software package or a web server during the period of this work. In addition to these two-class classification tools that distinguish PVPs and non-PVPs, other tools have been designed for identifying specific PVPs, such as the capsid and tail [5,11]. Distinct from these tools, PhANNs [8] is a multiclass classifier that not only can identify whether a given protein is PVP but also further classifies the predicted PVP into one of several major PVP classes, making PhANNs a more powerful tool for PVP annotation.

Although these tools achieved better performance than the other tools available prior to their publication, further efforts can be made to improve PVP annotation. For example, most of these tools were trained and tested using small-scale datasets containing fewer than 1,000 proteins, which may not reflect the full diversity of PVP sequence features. With the rapid growth of public databases, more PVPs and non-PVPs can be included in the algorithm development processes. In terms of the protein sequence characterization method used, most of these tools used amino acid composition-based vectors; for example, PhANNs uses a *k*-mer vector. However, such vectors may be sparse and thus difficult to fit by the algorithm. Additionally, for many of the currently available tools mentioned above, a feature selection step must be performed before the feature vector is imported into the algorithm. Moreover, except for PhANNs, the current PVP annotation tools can only distinguish PVPs and non-PVPs or identify a certain class of PVPs; they cannot further classify PVPs into specific classes, which prevents detailed analysis of the phage genome.

To improve the performance of PVP annotation, we present DeePVP. DeePVP takes a phage protein as input; then, the main module of DeePVP outputs a PVP likelihood score to reflect whether the given protein belongs to the PVP, while the extended module further calculates whether a predicted PVP belongs to one of ten major classes of PVPs, namely, head-tail joining, collar, tail sheath, tail fiber, portal, minor tail, major tail, baseplate, minor capsid, and major capsid, which are the dominant categories in PVP. Both modules use the one-hot encoding method to characterize the protein sequence and use a convolution neural network (CNN) as the classifier for protein feature extraction. Testing using the benchmark dataset and two application cases demonstrate the advantages of DeePVP over existing tools in PVP annotation.

Material and methods

Several benchmark training and testing datasets of PVP and non-PVP sequences have been constructed in previous work [8,11-23]. In this work, the benchmark dataset of PhANNs

(http://edwards.sdsu.edu/phanns/download/expandedDB.tgz) [8], which was constructed using proteins from the NCBI protein database and GenBank database, was used to develop DeePVP. This dataset was chosen for the following reasons: (1) at the time of this work, the PhANNs dataset is the largest PVP and non-PVP dataset, containing a total of 168,660 PVPs and 369,553 non-PVPs, whereas most of the other datasets contain less than 1,000 proteins; (2) the homology between the cross-validation and testing proteins is less than 40%, which is important in evaluating whether the algorithm can predict novel proteins, and within the cross-validation set, the sequence homology between the training and validation set in each rotation of the 10-fold cross validation is also less than 40%; and (3) the dataset includes all 10 major classes of PVPs that we focused on.

The framework of DeePVP is shown in Figure 1. Selecting an appropriate representation method for biological sequences is an important step for bioinformatics algorithm development. Although the *k*-mer frequency vector has been widely used in many studies, such global statistics may lose certain local sequence information, such as information related to conserved domains or motifs in the sequence [24]. In DeePVP, we used the “one-hot” encoding form to represent the protein sequence. In this way, each amino acid is represented by a “one-hot” vector containing 20 bits, in which 19 bits are 0 and a certain bit is 1; therefore, the information of each amino acid is retained in the digitized model (see Additional file 1 for more details). The “one-hot” encoded protein is first processed by the main module. The main module uses CNN to extract the sequence features to determine whether the given protein is a PVP. The CNN contains a 1D convolution layer, 1D global max pooling layer, batch normalization layer, full connection layer, and finally, a sigmoid layer that outputs a PVP score between 0 and 1. By default, a protein with a PVP score higher than 0.5 is regarded as a PVP. The extended module also uses CNN to classify the protein into a specific class of PVP. The CNN in the extended module contains a 1D convolution layer, 1D global max pooling layer, batch normalization layer, full connection layer, and finally, a softmax layer that outputs 10 likelihood scores representing the probability that the protein belongs to the head-tail joining, collar, tail sheath, tail fiber, portal, minor tail, major tail, baseplate, minor capsid, or major capsid

class. By default, the category with the highest score will be chosen as the final prediction. The details of the hyperparameter selection are provided in Additional file 1.

Figure 1. The framework of DeePVP. DeePVP takes a protein sequence as input, the main module calculates a PVP score representing the probability that the protein belongs to PVP, and the extended module calculates likelihood scores for each of the 10 PVP classes to determine the most likely class.

In the training process, both the PVPs and non-PVPs in the training set were used to train the main module, and only PVPs were used to train the extended module. In the prediction process, each protein is processed through the main module and extended module. However, it is worth noting that if a protein obtains a PVP score lower than the threshold, the 10 scores calculated by the extended module will not make sense because this protein is not a PVP. Therefore, in the DeePVP workflow, we normalize the 10 scores from the extended module such that their sum is equivalent to the PVP score.

Results

Performance comparison in the PVP identification task

We first evaluated the PVP identification performance of the main module of DeePVP using 10-fold cross validation with the cross-validation set. The evaluation criteria are as follows:

$$recall = \frac{TP}{TP + FN} \tag{1}$$

$$precision = \frac{TP}{TP + FP} \tag{2}$$

$$F1 - score = 2 \times \frac{recall \times precision}{recall + precision} \tag{3}$$

where *TP*, *TN*, *FN* and *FP* represent the number of true positive, true negative, false negative and false positive predictions, respectively. Among these three criteria, the *F1-score* can serve as a comprehensive index for evaluating PVP identification tools. We found that the main module of DeePVP achieved

satisfactory performance, with average *recall*, *precision* and *F1-score* values of 85.61%, 96.65% and 90.76%, respectively.

We then trained the main module of DeePVP with all sequences in the cross-validation set and compared the performance between DeePVP and several state-of-the-art tools, namely, PVPred, PVP-SVM, PVPred-SCM, Meta-iPVP, PhANNs and VirionFinder, using the test set. The performance comparison is shown in Table 1. Although the *recall* of DeePVP is slightly lower than that of PhANNs and VirionFinder, the *precision* of DeePVP is much better than those of all the other tools, and the comprehensive index of *F1-score* is 9.05% much higher than that of PhANNs, which performs the best among the other tools. Since DeePVP and PhANNs were trained using the same training data, the improved performance of DeePVP suggests that "one-hot" encoding may provide a more detailed representation method than the *k*-mer frequency used to characterize the protein sequences in PhANNs, and the deep convolution neural network in DeePVP may be more powerful than the simple shallow network containing 2 hidden layers used for feature extraction in PhANNs.

Table 1. Performance comparison of DeePVP and related tools in the PVP identification task.

Performance comparison in the PVP classification task

We further evaluated the PVP classification performance of the extended module of DeePVP. For each PVP category, we used the criteria of *recall*, *precision* and *F1-score* to evaluate the performance of the tool, and we also used the *accuracy*, which was defined as the ratio of the correctly predicted sequences to the total number of sequences, to evaluate the overall performance of the tool. In the 10-fold cross validation, the extended module of DeePVP again achieved satisfactory performance, with an average *accuracy* of 90.19%. The *recall*, *precision* and *F1-score* for each PVP category in the cross validation are shown in Table S1 of Additional file 1.

After training the extended module of DeePVP using all sequences in the cross validation set, we further compared the performance between the extended module of DeePVP and PhANNs in the PVP

classification task using the test set. Since the other tools cannot further classify a given PVP into a specific class, these tools were not included in this analysis. During the comparison, we assume that all the PVPs have already been correctly predicted in the upstream analysis; therefore, non-PVPs are excluded from the test set in this subsection. In practice, in the released package of DeePVP, the main module and the extended module can be run consecutively using an integrated pipeline or be run separately. For example, if researchers have already identified PVPs using other computational or experimental methods, such as mass spectrometry, they can run the extended module of DeePVP directly for PVP classification.

We used the criteria of *recall*, *precision* and *F1-score* to evaluate the performance of DeePVP and PhANNs for each PVP category. We also used *accuracy* to evaluate the overall performance of the tools. The performance comparison is shown in the Table 2. The overall *accuracy* of DeePVP is 3.72% higher than that of PhANNs, indicating that DeePVP has a better ability to classify PVPs. In terms of each category, although the *F1-score* values of DeePVP for the major tail, tail fiber, tail sheath and collar are lower than those of PhANNs, DeePVP presents a higher *F1-score* for other categories. While the *accuracy* advantage of DeePVP is not as prominent, as it is only 3.72% higher than that of PhANNs, it is worth noting that such performance is evaluated under the assumption that all PVPs have been correctly predicted. Since DeePVP performs much better than PhANNs in PVP identification, we consider that the comprehensive PVP annotation performance of DeePVP is better than that of PhANNs.

Table 2. Performance comparison of DeePVP and PhANNs in the PVP classification task.

Application case 1: PVP annotation of the mycobacteriophage PDRPxv genome

To demonstrate the value and reliability of DeePVP in PVP annotation, we first used DeePVP and related tools to perform PVP annotation of the genome of the mycobacteriophage PDRPxv (GenBank accession: KR029087), which is considered a candidate therapeutic for pathogenic *Mycobacterium* species [25]. The

PVPs in the PDRPxv genome have been identified experimentally by mass spectrometry; such experimental data can be used to evaluate the reliability of computational tools. The tools DeePVP, PVPred, PVP-SVM, PVPred-SCM, Meta-iPVP, PhANNs and VirionFinder were used to perform PVP annotation of the PDRPxv genome, and the mass spectrometry data were used to evaluate the *recall*, *precision* and *F1-score* of each tool. The performance of each tool in the PVP identification task is shown in Table 3. We found that most of the compared tools did not perform well, with *F1-scores* lower than 50%. PhANNs performed better than the other tools, and the *F1-score* of DeePVP was 21.19% much higher than that of PhANNs, indicating that DeePVP provides a more reliable prediction.

Table 3. Performance comparison in the PVP identification task in the PDRPxv genome, validated according to mass spectrometry data.

In Figure 2, we show the base coordinates for the PVPs uncovered by mass spectrometry and the PVPs predicted by each tool. The mass spectrometry data showed that all PVPs were located within a compact PVP-enriched region, and no PVPs were identified outside the PVP-enriched region. In fact, it has been shown that PVPs are often located near each other within the genome [26,27], and such PVP distribution patterns may be common among phages. Interestingly, we found that the PVP distribution pattern revealed by DeePVP was quite consistent with that revealed by mass spectrometry. Although DeePVP failed to predict a few PVPs, all of the other predicted PVPs were located within the PVP-enriched region. In contrast, the PVPs predicted by the other tools seem to be distributed randomly throughout the genome. This phenomenon shows that, in comparison with other tools, DeePVP provides more authentic prediction and has a better ability to reveal the genomic structure of a phage genome.

Figure 2. The distribution of the PVPs uncovered by mass spectrometry and the PVPs predicted by each tool on the mycobacteriophage PDRPxv genome. The blue bars represent the PDRPxv genome, and the red boxes represent the PVPs. The horizontal axis represents the base coordinates.

According to mass spectrometry, the PDRPxv genome contains 12 PVPs. Sinha *et al*. [25] inferred the putative function of each PVP through a combination of several strategies, including domain search, homology analysis, adjacent gene analysis and protein secondary structure analysis. In Table 4, we compared the PVP classes predicted by DeePVP and PhANNs with the putative functions revealed by Sinha *et al*. We found that the predictions of DeePVP and PhANNs were basically consistent with the putative functions. In particular, 5 putative minor tail proteins are encoded continuously (Gp29-Gp33) in the genome, and both DeePVP and PhANNs predicted this minor tail protein cluster appropriately.

Table 4. PDRPxv genome PVP classification by DeePVP and PhANNs.

Application case 2: A novel conserved PVP-enriched region was found in *Escherichia* phage HP3

Viral genomes are highly diverse, which presents a challenge to understanding viral evolutionary mechanisms [28]. Phage diversity is sometimes driven by PVPs [11]. In this subsection, we used DeePVP to perform PVP annotation on *Escherichia* phage HP3 (RefSeq accession: NC_041920.1), a phage that may be an effective treatment for murine models of bacteraemia [29], to reveal its genomic features. According to the annotation of the RefSeq database, the *Escherichia* phage HP3 genome encodes 269 proteins, all of which lack functional annotation information in the RefSeq database. Additionally, no experimental data are available to determine which proteins are PVPs; therefore, PVP annotation using computational methods is an efficient way to analyse the genome. We used all the related tools to perform PVP annotation on the phage HP3 genome. The base coordinates for the PVPs predicted by each tool are shown in Figure 3. We defined a “PVP prediction reliability index” for each PVP predicted by each tool to evaluate the prediction reliability. For a certain protein predicted as a PVP by a certain tool, if this protein was also predicted as a PVP by n other tools, then the “reliability index” for this predicted PVP

was *n*. A higher *n* indicates that this protein is predicted as PVP by more tools at the same time, and therefore, the prediction may be more reliable. The average “reliability index” of each tool is shown in Figure 3. We found that DeePVP achieved the highest index of 4.75, indicating that DeePVP might produce the most reliable predictions.

Additionally, we found that the genomic features of the phage HP3 genome revealed by DeePVP were interesting. Compared with the PVP distributions of other tools, which were disperse across the genome, the distribution of all PVPs predicted by DeePVP was highly compact; a PVP-enriched region was clearly present, and there were no predicted PVPs outside the PVP-enriched region. This phenomenon is quite consistent with that of mycobacteriophage PDRPxv in application case 1, in which all PVPs are compactly encoded on the genome. Since such compact PVP distribution patterns are common [26,27], we consider that DeePVP may perform better in revealing the features of the phage genome. Meanwhile, from the DeePVP prediction results, we found that there was a clear spacer without predicted PVPs within the PVP-enriched region, as shown in Figure 3.

Figure 3. The distribution of PVPs predicted by each tool in the *Escherichia* phage HP3 genome. The blue bars represent the *Escherichia* phage HP3 genome, and the red boxes represent the predicted PVPs. The average reliability index of each tool is shown on the right side of the bar. The genomic features revealed by DeePVP are also marked in the first bar. The horizontal axis represents the base coordinates.

To perform further comparative genomic analysis, we aligned the phage HP3 genome to the NT database using the NCBI blastn online server (https://blast.ncbi.nlm.nih.gov/Blast.cgi), with all parameters at the default settings. The alignment results of the top 100 subject sequences are shown in Figure 4, and detailed information about each subject sequence obtained from the server is provided in Additional file 2. All subject sequences were phage sequences. The alignment graph showed that the phage HP3 genome contained a low conserved region, which was conserved in some phage genomes but

lacked obvious homology in other genomes, and a highly conserved region, which was present in almost all subject sequences. In addition, within the highly conserved region, there was a low conserved spacer that was less conserved among the subject sequences. More interestingly, when comparing the DeePVP prediction in Figure 3 with the alignment results in Figure 4, we found that the highly conserved region corresponded to the PVP-enriched region, while the low conserved region corresponded to the region outside of the PVP-enriched region. Additionally, the low conserved spacer within the highly conserved region approximately corresponded to the spacer within the PVP-enriched region. The above phenomenon suggests that the *Escherichia* phage HP3 genome contains a compact PVP-enriched region that has been conserved during viral evolution and that this PVP-enriched region contains a non-PVP spacer that might have been generated through recombination or horizontal gene transfer.

Figure 4. The alignment results of the top 100 subject sequences for the *Escherichia* phage HP3 genome. The blue bar represents the phage HP3 genome query sequence. Each line below the query sequence represents a certain subject sequence, and the coloured box on the subject sequence represents a certain blast hit.

Moreover, the alignment results showed that most of the subject sequences (71% in total, including the phage HP3 genome itself) were also *Escherichia* phages, as shown in Figure 5. Such results indicate that among phages that infect the same host, the PVP-enriched region may be more conserved than the non-PVP region during the viral evolution process. This phenomenon is easy to interpret because the host of the phage is determined by the interaction between the PVPs and the host cell receptors [30], and therefore, phages that infect the same host genus may have similar PVPs. It is thus expected that DeePVP will be a useful tool for phage host prediction by revealing the PVP biomarkers that are conserved during the viral evolution process and for selecting a suitable phage that infects the specific bacterium in phage therapy.

Figure 5. Host genus composition of the subject sequences. We calculated the frequency of each host genus among the alignment results of *Escherichia* phage HP3 and found that most of the subject sequences were also *Escherichia* phages. The frequency of each host genus is shown in brackets.

The PVP classification results of DeePVP and PhANNs are shown in Table 5 and Table S2 of Additional file 2, respectively. We found that the categories predicted by DeePVP covered most of the essential PVPs for the viral particle, including the major capsid, major tail, tail sheath, tail fiber, baseplate and portal. Additionally, three tail-associated PVPs, tail fibers, are encoded next to each other (YP_009965877.1, YP_009965879.1, YP_009965880.1). This phenomenon is similar to that of application case 1, in which five putative tail-associated PVPs, minor tails, are encoded continuously in the genome.

Table 5. PVP categories predicted by DeePVP in the *Escherichia* phage HP3 genome.

Discussion

To develop DeePVP, we chose to train a two-class classifier and a ten-class classifier separately rather than to train an eleven-class classifier directly. This is because the number of protein sequences in each category of the dataset is unbalanced. For example, in the dataset, 369,553 of the proteins belong to the non-PVP category, while only 2,105 proteins belong to the collar category. An unbalanced dataset presents a challenge for training a neural network because the neural network may tend to assign most of the samples to the category with the largest size automatically. In DeePVP, we first trained a CNN to separate PVP and non-PVP sequences; this reduces the impact of the non-PVP category, which contains many more sequences than the other PVP categories, on the PVP classification task.

Among the related tools, amino acid composition-based feature vectors are commonly used to characterize sequences. Such global statistics may fail to capture certain local information, such as

conserved domains or motifs, in the sequence. Additionally, such feature vectors may be sparse. For example, PhANNs uses a *k*-mer-based feature vector containing thousands of bits, while a large number of proteins contain only approximately 250 aa; therefore, most of the bits in the feature vector are likely to be 0. Such sparse vectors may make it difficult for the algorithm to fit the data. In DeePVP, we used "one-hot" encoding for the sequence, such that the information about each amino acid was retained in the characterization model. We then used CNN to extract the useful features from the raw data. It has been shown that CNN is powerful for extracting useful features, and the convolution kernel may serve as a sensitive position weight matrix (PWM) to detect local specific motifs [31-33]. We therefore consider that this method may help to improve the performance of DeePVP.

Although the benchmark dataset we used in this work is by far the largest and best designed PVP and non-PVP dataset currently available, it is primarily focused on the 10 major classes of PVPs and was created using keyword searches of public databases. Thus, some less frequently occurring PVPs may be excluded from the dataset, and thus DeePVP may fail to identify some types of PVPs. For example, in application case 1, DeePVP failed to identify a putative scaffolding protein (Gp11) and a putative tape measure protein (Gp28), which may be because these two types of proteins were not included in the dataset by the keyword search process. Therefore, further efforts should be made to create more exhaustive PVP sets to further improve the algorithm performance.

Although the comprehensive index *F1-score* of the main module of DeePVP is much better than that of the other tools, the *recall* of DeePVP is slightly lower than that of some tools, such as PhANNs and VirionFinder, in the PVP identification task, indicating that DeePVP may fail to identify some PVPs. On the other hand, DeePVP was better able to identify the PVP-enriched region, and PVPs are encoded compactly within a single region in many phages. Therefore, we suggest that users can combine DeePVP with other tools to identify as many PVPs as possible with a low false-positive prediction rate. In application case 1, for example, the user can first use DeePVP to identify the PVP-enriched region (from Gp8 to Gp33, as shown in Figure 2 and Table 4). Then, within this region, users can apply the more sensitive PhANNs to identify more PVPs. Since proteins within the region are more likely to be PVPs,

such an operation may be less likely to generate false positive predictions. With this approach, the PVP of Gp28, which fails to be predicted by DeePVP, was included in the prediction, and the *recall* was increased from 75% to 83.33%. Although this approach also introduced three false positive predictions (Gp14, Gp19, Gp26), a large number of false positive predictions outside the PVP-enriched region are excluded. In the future, it would be worth constructing a comprehensive workflow that integrates different algorithms to improve the PVP annotation performance.

Conclusion

In this work, we present DeePVP, a new tool for PVP annotation of a phage genome. The main module of DeePVP aims to identify whether a phage protein is a PVP, while the extended module can further judge the class to which the PVP belongs. Evaluation using the large-scale benchmark dataset shows that DeePVP performs much better than the related tools. We provided two application cases to demonstrate the value of DeePVP. In the case of mycobacteriophage PDRPxv, by referring to experimental mass spectrometry data, we illustrated that the prediction of DeePVP was more reliable and could better reveal the compact distribution pattern of PVP over the genome than those of other tools. We then used DeePVP to perform PVP prediction on *Escherichia* phage HP3, which previously lacked experimental data and annotation information for its PVPs. Compared with the other tools, DeePVP again showed a clear PVP-enriched region within the genome, and we found that this newly discovered PVP-enriched region is conserved in many other phages that infect the same host genus during the viral evolution process. We therefore suggest that DeePVP may be a powerful tool for various phage genomic analysis applications, such as host prediction. DeePVP software is optimized in both a virtual machine with graphical user interface (GUI) and a docker, which makes the software easy to install on a local PC or high-performance computing (HPC) system. Non-computer professionals who are not familiar with the command line can also easily run the tool on large-scale data.

Availability of supporting source code and requirements

Project name: DeePVP.

Project home page: https://github.com/fangzcbio/DeePVP/.

Operating system: The code of DeePVP was written on Linux. We optimized the program in a virtual machine and docker thus DeePVP is platform independent.

Programming language: python, matlab.

Other requirements: no other requirements are needed.

License: GPL-3.0.

Availability of supporting data

The related supporting data of the manuscript can be downloaded from https://github.com/fangzcbio/DeePVP/releases/download/Supporting_data/data.zip.

Additional file

Additional file 1: (i) description of the one-hot encoding form, (ii) description of the details of the hyperparameter selection of the neural network, (iii) Table S1. The average of *recall*, *precision*, *F1-score* and *accuracy* of the extended module of DeePVP in the 10-fold cross validation, and (iv) Table S2. PVP category predicted by PhANNs on the *Escherichia* phage HP3 genome.

Additional file 2: detailed information about each subject sequence of *Escherichia* phage HP3 genome obtained from the NCBI blastn server.

List of abbreviations

PVP: phage virion protein

CNN: convolution neural network

GUI: graphical user interface

HPC: high-performance computing

Ethics approved and consent to participate

Not applicable

Consent for publication

Not applicable

Competing interests

The authors declare that they have no competing interests

Funding

This investigation was financially supported by the National Natural Science Foundation of China (82102508, 81925026, 82002201, 81800746).

Authors' contributions

ZCF and HWZ proposed and designed the study. ZCF and TF constructed the data sets, and wrote and optimized the code. ZCF and MXC wrote and revised the manuscript and all authors proofread and improved the manuscript.

References

[1] Güemes AGC, Youle M, Cantú VA, *et al*. Viruses as Winners in the Game of Life. *Annu Rev Virol* 2016; **3**: 197-214.

[2] Edwards RA, Rohwer F. Viral metagenomics. *Nat Rev Microbiol* 2005; **3**(6): 504–10.

[3] Hayes S, Mahony J, Nauta A, *et al*. Metagenomic approaches to assess bacteriophages in various environmental niches. *Viruses* 2017; **9**(6): 127.

[4] Meng C, Zhang J, Ye X, *et al*. Review and comparative analysis of machine learning-based phage virion protein identification methods. *Biochim Biophys Acta Proteins Proteom* 2020; **1868**(6): 140406.

[5] Galiez C, Magnan CN, Coste F, *et al*. VIRALpro: a tool to identify viral capsid and tail sequences. *Bioinformatics* 2016; **32**(9): 1405-7.

[6] Boeckaerts D, Stock M, Criel B, *et al*. Predicting bacteriophage hosts based on sequences of annotated receptor-binding proteins. *Sci Rep* 2021; **11**(1): 1467.

[7] Lekunberri I, Subirats J, Borrego CM, *et al*. Exploring the contribution of bacteriophages to antibiotic resistance. *Environ Pollut* 2017; **220**(Pt B): 981-4.

[8] Cantu VA, Salamon P, Seguritan V, *et al*. PhANNs, a fast and accurate tool and web server to classify phage structural proteins. *PLoS Comput Biol* 2020; **16**(11): e1007845.

[9] Roux S, Enault F, Hurwitz BL, *et al*. VirSorter: mining viral signal from microbial genomic data. *PeerJ* 2015; **3**: e985.

[10] Lavigne R, Ceyssens PJ, Robben J. Phage proteomics: applications of mass spectrometry. *Methods Mol Biol* 2009; **502**: 239-51.

[11] Seguritan V, Alves N Jr, Arnoult M, *et al*. Artificial neural networks trained to detect viral and phage structural proteins. *PLoS Comput Biol* 2012; **8**(8): e1002657.

[12] Feng PM, Ding H, Chen W, *et al*. Na ïve Bayes classifier with feature selection to identify phage virion proteins. *Comput Math Methods Med* 2013; **2013**:530696.

[13] Ding H, Feng PM, Chen W, *et al*. Identification of bacteriophage virion proteins by the ANOVA feature selection and analysis. *Mol Biosyst* 2014; **10**(8): 2229-35.

[14] Zhang L, Zhang C, Gao R, *et al*. An ensemble method to distinguish bacteriophage virion from non-virion proteins based on protein sequence characteristics. *Int J Mol Sci* 2015; **16**(9): 21734-58.

[15] Manavalan B, Shin TH, Lee G. PVP-SVM: sequence-based prediction of phage virion proteins using a support vector machine. *Front Microbiol* 2018; **9**: 476.

[16] Pan Y, Gao H, Lin H, *et al*. Identification of bacteriophage virion proteins using multinomial naive Bayes with g-gap feature tree. *Int J Mol Sci* 2018; **19**(6): 1779.

[17] Tan JX, Dao FY, Lv H, *et al*. Identifying phage virion proteins by using two-step feature selection methods. *Molecules* 2018; **23**(8): 2000.

[18] Ru X, Li L, Wang C. Identification of phage viral proteins with hybrid sequence features. *Front Microbiol* 2019; **10**: 507.

[19] Arif M, Ali F, Ahmad S, *et al*. Pred-BVP-Unb: Fast prediction of bacteriophage Virion proteins using un-biased multi-perspective properties with recursive feature elimination. *Genomics* 2020; **112**(2): 1565-74.

[20] Charoenkwan P, Kanthawong S, Schaduangrat N, *et al*. PVPred-SCM: improved prediction and analysis of phage virion proteins using a scoring card method. *Cells* 2020; **9**(2): 353.

[21] Charoenkwan P, Nantasenamat C, Hasan MM, *et al*. Meta-iPVP: a sequence-based meta-predictor for improving the prediction of phage virion proteins using effective feature representation. *J Comput Aided Mol Des* 2020; **34**(10): 1105-16.

[22] Fang Z, Zhou H. VirionFinder: identification of complete and partial prokaryote virus virion protein from virome data using the sequence and biochemical properties of amino acids. *Front Microbiol* 2021; **12**:615711.

[23] Han H, Zhu W, Ding C, *et al*. iPVP-MCV: A Multi-Classifier Voting Model for the Accurate Identification of Phage Virion Proteins. *Symmetry* 2021; **13**(8): 1506.

[24] Fang Z, Tan J, Wu S, *et al*. PPR-Meta: a tool for identifying phages and plasmids from metagenomic fragments using deep learning. *GigaScience* 2019; **8**(6), giz066.

[25] Sinha A, Eniyan K, Manohar P, *et al*. Characterization and genome analysis of B1 sub-cluster mycobacteriophage PDRPxv. *Virus Res* 2020; **279**: 197884.

[26] Hatfull GF. Molecular genetics of mycobacteriophages. *Microbiol Spectr* 2014; **2**(2): 81-119.

[27] Sassi M, Bebeacua C, Drancourt M, *et al*. The first structure of a mycobacteriophage, the Mycobacterium abscessus subsp. bolletii phage Araucaria. *J Virol* 2013; **87**(14): 8099-109.

[28] Dion MB, Oechslin F, Moineau S. Phage diversity, genomics and phylogeny. *Nat Rev Microbiol* 2020; **18**(3):125-38.

[29] Green SI, Kaelber JT, Ma L, *et al*. Bacteriophages from ExPEC reservoirs kill pandemic multidrug-resistant strains of clonal group ST131 in animal models of bacteremia. *Sci Rep* 2017 **7**:46151.

[30] Stone E, Campbell K, Grant I, *et al*. Understanding and Exploiting Phage-Host Interactions. *Viruses* 2019; **11**(6):567.

[31] Alipanahi B, Delong A, Weirauch MT, *et al*. Predicting the sequence specificities of DNA- and RNA-binding proteins by deep learning. *Nat Biotechnol* 2015; **33**(8):831-8.

[32] Kelley DR, Snoek J, Rinn JL. Basset: learning the regulatory code of the accessible genome with deep convolutional neural networks. *Genome Res* 2016; **26**(7):990-9.

[33] Zhou J, Troyanskaya OG. Predicting effects of noncoding variants with deep learning-based sequence model. *Nat Methods* 2015; **12**(10):931-4.

Table 1. Performance comparison of DeePVP and related tools in the PVP identification task.

Tool	*Recall* (%)	*Precision* (%)	*F1-score* (%)
**DeePVP**	**88.10**	**96.75**	**92.22**
VirionFinder	90.91	44.00	59.30
PhANNs	91.68	76.11	83.17
Meta-iPVP	82.41	53.29	64.72
PVPred-SCM	41.28	38.71	39.95
PVP-SVM	41.31	46.19	43.62
PVPred	29.84	42.23	34.97

Table 2. Performance comparison of DeePVP and PhANNs in the PVP classification task.

Category	Tool	*Recall* (%)	*Precision* (%)	*F1-score* (%)	*Accuracy* (%)
Major capsid	DeePVP	98.58	90.88	94.57	NA
	PhANNs	91.98	94.24	93.10	
Minor capsid	DeePVP	50.62	44.09	47.13	
	PhANNs	80.25	13.98	23.81	
Baseplate	DeePVP	86.49	95.96	90.98	
	PhANNs	78.85	88.76	83.51	
Major tail	DeePVP	77.09	61.23	68.25	
	PhANNs	80.48	86.88	83.56	
Minor tail	DeePVP	90.28	93.24	91.74	
	PhANNs	85.42	90.50	87.89	
Portal	DeePVP	93.10	93.62	93.36	
	PhANNs	85.88	93.77	89.65	
Tail fiber	DeePVP	78.24	68.42	73.00	
	PhANNs	77.93	69.66	73.56	
Tail sheath	DeePVP	92.86	99.42	96.03	
	PhANNs	94.39	99.64	96.94	
Collar	DeePVP	36.33	80.15	50.00	
	PhANNs	87.00	75.65	80.93	
Head-tail joining	DeePVP	96.48	96.33	96.40	
	PhANNs	88.49	71.88	79.33	
**All**	**DeePVP**		NA		**91.06**
	PhANNs				87.34

Table 3. Performance comparison in the PVP identification task in the PDRPxv genome, validated according to mass spectrometry data.

Tool	*Recall* (%)	*Precision* (%)	*F1-score* (%)
**DeePVP**	**75.00**	**100.00**	**85.71**
VirionFinder	91.67	27.50	42.31
PhANNs	83.33	52.63	64.52
Meta-iPVP	75.00	15.79	26.09
PVPred-SCM	58.33	12.28	20.29
PVP-SVM	58.33	17.07	26.42
PVPred	50.00	18.18	26.67

Table 4. PDRPxv genome PVP classification by DeePVP and PhANNs.

Protein ID	Putative function[a]	DeePVP prediction	PhANNs prediction
Gp8	Portal protein	Portal	Portal
Gp10	Minor head protein	Portal	Portal
Gp11	Scaffolding protein	NA[b]	NA
Gp12	Major capsid protein	Major capsid	Major capsid
Gp18	Major tail subunit	Major tail	Major tail
Gp25	Tail assembly chaperone	NA	NA
Gp28	Tape measure protein	NA	Minor tail
Gp29	Minor tail protein	Minor tail	Minor tail
Gp30	Minor tail protein	Minor tail	Minor tail
Gp31	Minor tail protein	Minor tail	Minor tail
Gp32	Minor tail protein	Minor tail	Minor tail
Gp33	Minor tail protein	Minor tail	Minor tail

a: Putative function as analysed by Sinha *et al*. [25]; b: NA indicates that the tool did not identify the corresponding protein as a PVP.

Table 5. PVP category predicted by DeePVP on the *Escherichia* phage HP3 genome.

Protein ID	DeePVP prediction
YP_009965784.1	Tail sheath
YP_009965791.1	Baseplate
YP_009965797.1	Tail fiber
YP_009965805.1	Tail sheath
YP_009965807.1	Portal
YP_009965812.1	Major capsid
YP_009965814.1	Major capsid
YP_009965818.1	Major tail
YP_009965826.1	Baseplate
YP_009965877.1	Tail fiber
YP_009965879.1	Tail fiber
YP_009965880.1	Tail fiber

Figure 1
Click here to access/download;Figure;Figure 1.pdf

Figure 1

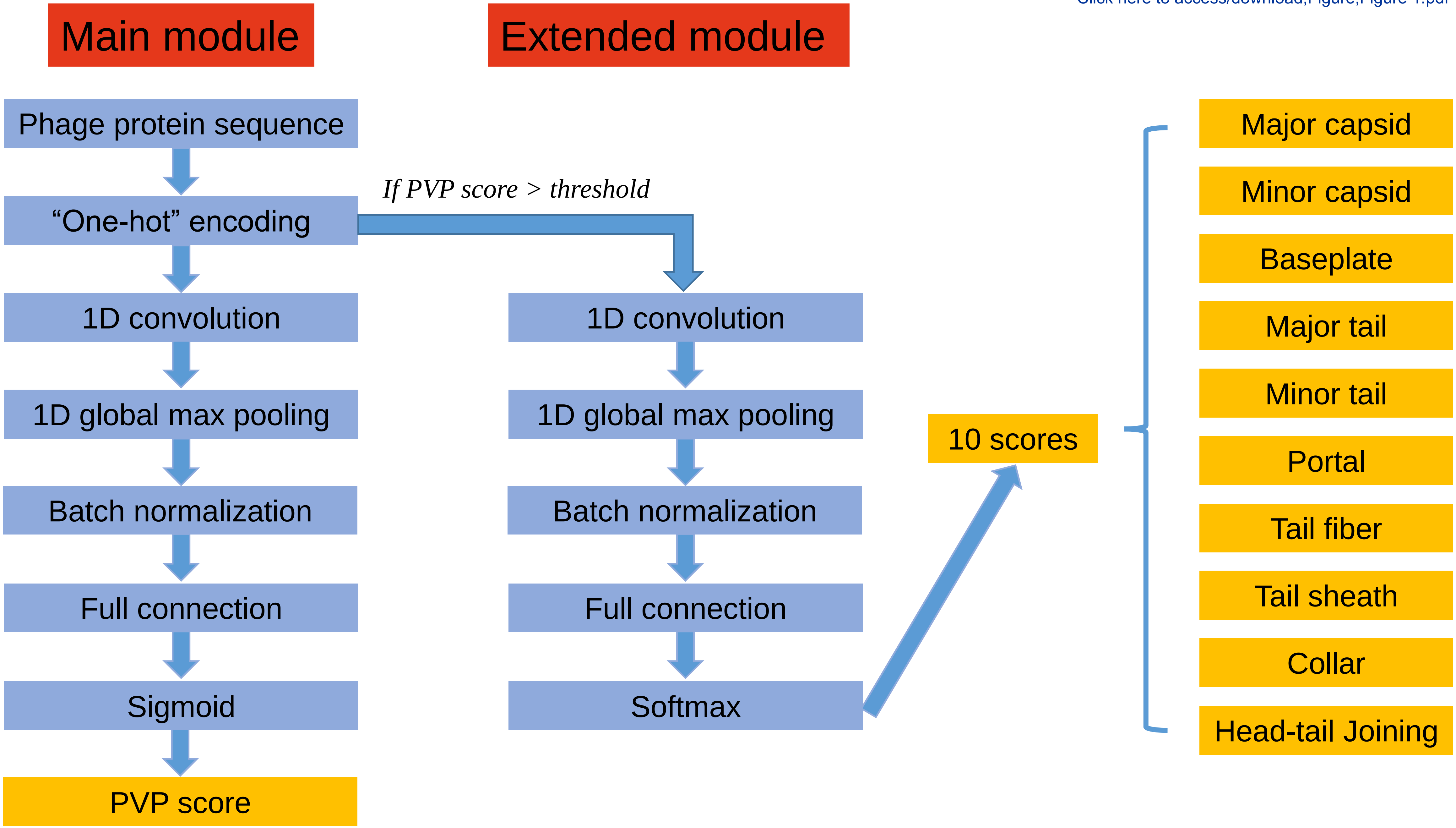

Click here to access/download;Figure;Figure 2.pdf

Figure 2

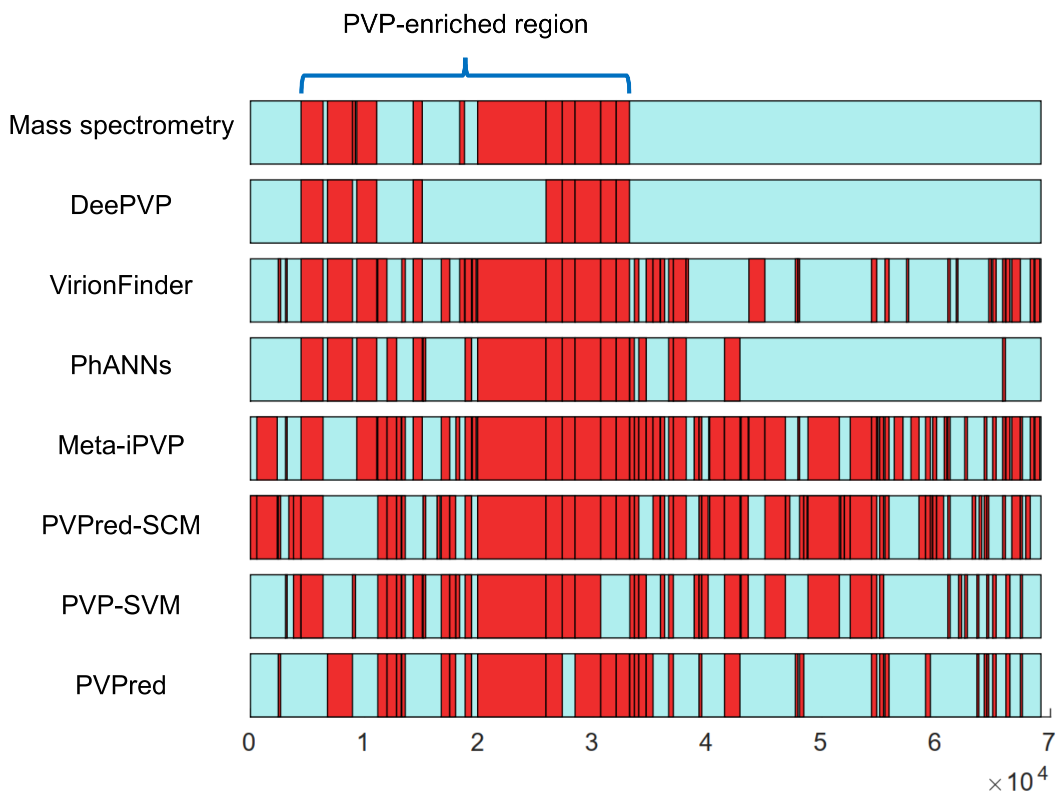

Figure 3

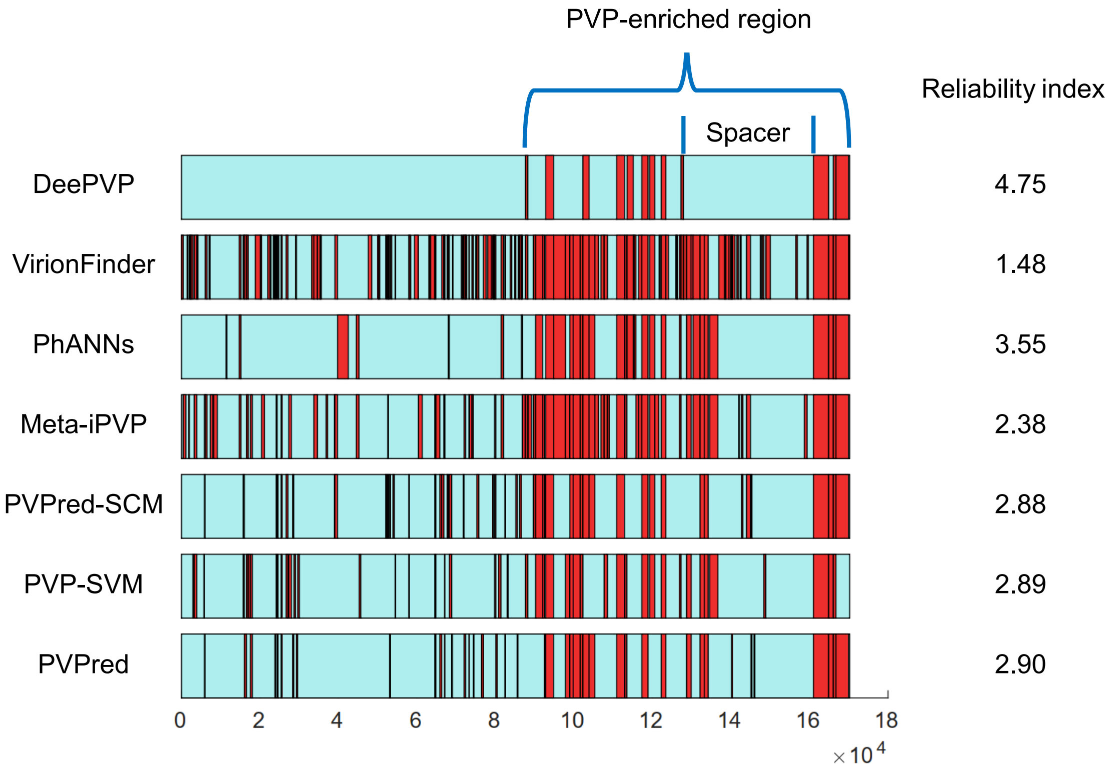
PVP-enriched region
Spacer
Reliability index
DeePVP
VirionFinder
PhANNs
Meta-iPVP
PVPred-SCM
PVP-SVM
PVPred
4.75
1.48
3.55
2.38
2.88
2.89
2.90
0
2
4
6
8
10
12
14
16
18
×10^4

Click here to access/download;Figure;Figure 4.pdf

Figure 4

Distribution of the top 3,739 Blast Hits of *Escherichia* phage HP3 on 100 subject sequences in the NT database

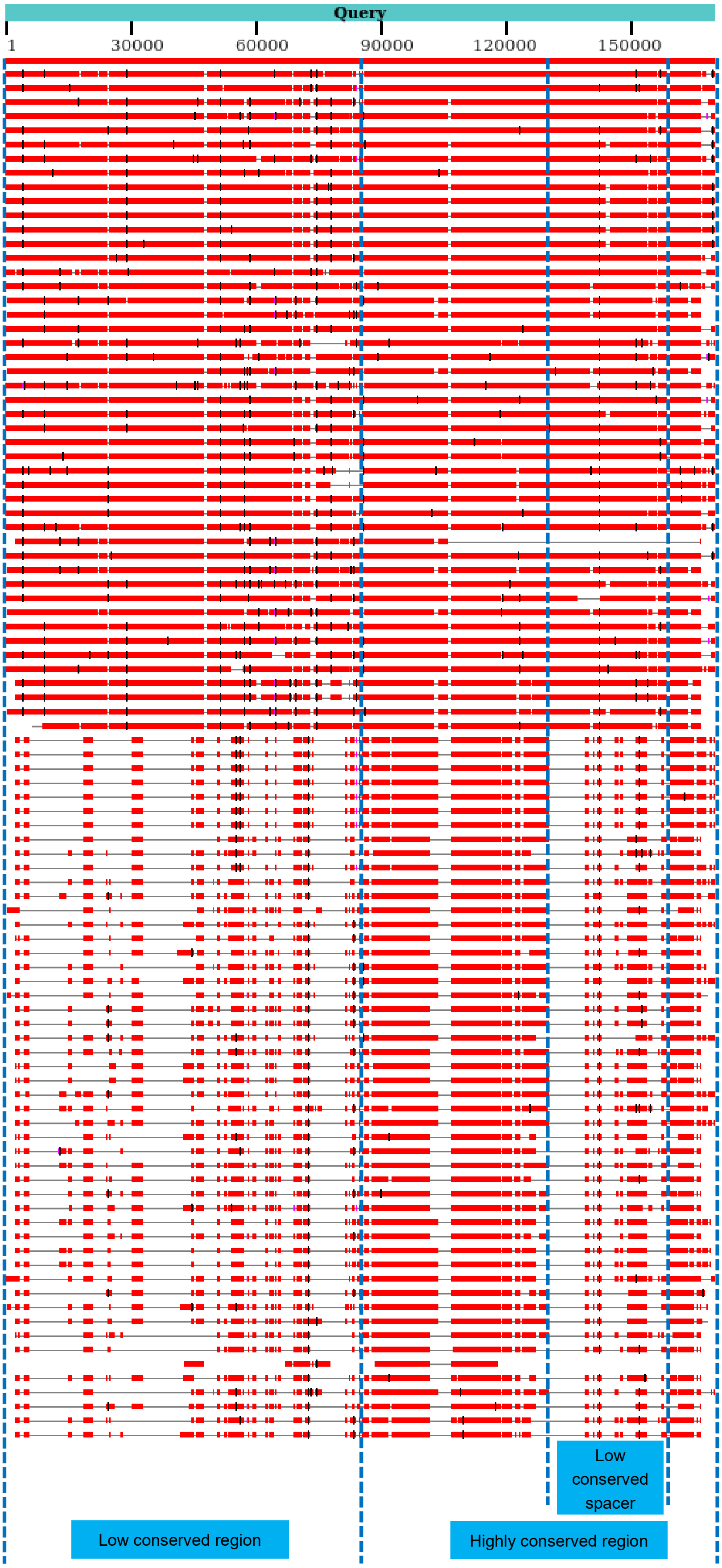

Figure 5

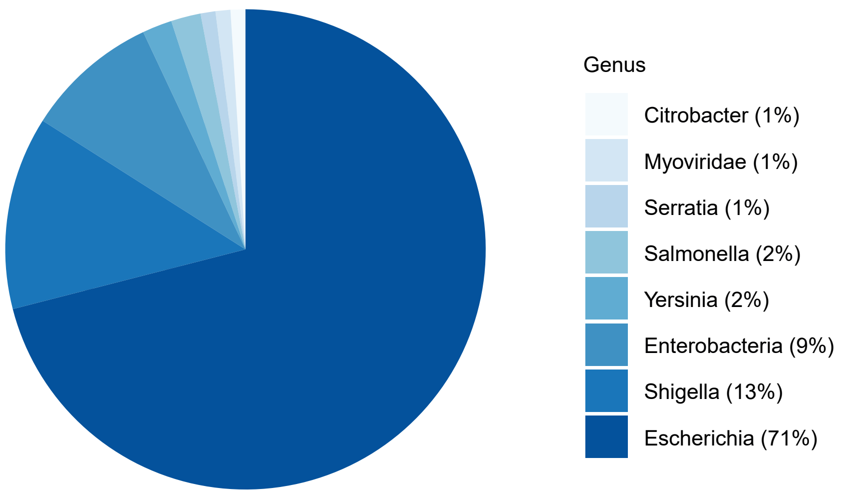
Genus
Citrobacter (1%)
Myoviridae (1%)
Serratia (1%)
Salmonella (2%)
Yersinia (2%)
Enterobacteria (9%)
Shigella (13%)
Escherichia (71%)

Additional file 1

Click here to access/download
Supplementary Material
Additional file 1.docx

Additional file 2

Click here to access/download
Supplementary Material
Additional file 2.txt
