# Supplementary material for: DeePVP: Identification and classification of phage virion proteins using deep learning
Source: Gigascience. 2022 Aug 11;11:giac076. doi: 10.1093/gigascience/giac076 (PMC9366990; doi:10.1093/gigascience/giac076)
Supplement: giac076_Supplemental_Files [file giac076_supplemental_files.zip › Additional file 1.pdf]

1 “One-hot” encoding form

In DeePVP, each amino acid is represented by a “one-hot” vector with 20 bits, as described below:

A: [0 0 0 0 0 0 0 0 0 0 0 0 0 0 0 0 0 0 0 1]
C: [0 0 0 0 0 0 0 0 0 0 0 0 0 0 0 0 0 0 1 0]
D: [0 0 0 0 0 0 0 0 0 0 0 0 0 0 0 0 0 1 0 0]
E: [0 0 0 0 0 0 0 0 0 0 0 0 0 0 0 0 1 0 0 0]
F: [0 0 0 0 0 0 0 0 0 0 0 0 0 0 0 1 0 0 0 0]
G: [0 0 0 0 0 0 0 0 0 0 0 0 0 0 1 0 0 0 0 0]
H: [0 0 0 0 0 0 0 0 0 0 0 0 0 1 0 0 0 0 0 0]
I: [0 0 0 0 0 0 0 0 0 0 0 0 1 0 0 0 0 0 0 0]
K: [0 0 0 0 0 0 0 0 0 0 0 1 0 0 0 0 0 0 0 0]
L: [0 0 0 0 0 0 0 0 0 0 1 0 0 0 0 0 0 0 0 0]
M: [0 0 0 0 0 0 0 0 0 1 0 0 0 0 0 0 0 0 0 0]
N: [0 0 0 0 0 0 0 0 1 0 0 0 0 0 0 0 0 0 0 0]
P: [0 0 0 0 0 0 0 1 0 0 0 0 0 0 0 0 0 0 0 0]
Q: [0 0 0 0 0 0 1 0 0 0 0 0 0 0 0 0 0 0 0 0]
R: [0 0 0 0 0 1 0 0 0 0 0 0 0 0 0 0 0 0 0 0]
S: [0 0 0 0 1 0 0 0 0 0 0 0 0 0 0 0 0 0 0 0]
T: [0 0 0 1 0 0 0 0 0 0 0 0 0 0 0 0 0 0 0 0]
V: [0 0 1 0 0 0 0 0 0 0 0 0 0 0 0 0 0 0 0 0]
W: [0 1 0 0 0 0 0 0 0 0 0 0 0 0 0 0 0 0 0 0]
Y: [1 0 0 0 0 0 0 0 0 0 0 0 0 0 0 0 0 0 0 0]

For a given protein, the “one-hot” vectors of each amino acid are joined together according to the amino acid sequence order. Therefore, a protein with length L can be represented by a “one-hot” matrix of length L and width 20. The convolutional neural network needs to fix the size of the input matrix. We found that 99.83% of the proteins in the benchmark dataset were shorter than 2000 aa. To save computational resources, we exclude the amino acids downstream of the 2000th aa. For sequences shorter than 2000 aa, the “one-hot” matrix is padded with zero vectors to 2000 rows.

2 Hyperparameter selection for the convolutional neural network

2.1 Main module

Hyperparameters of the 1D convolution layer: the length of the convolution layer was set to 9, the number of convolutional kernels was set to 700, and the “ReLU” activation function was used.
Hyperparameters of the full connection layer: the number of nodes was set to 700, and the “ReLU” activation function was used.
Other hyperparameters: In the training process, two dropout layers were deposited between the batch normalization layer and the full connection layer and between the full connection layer and the sigmoid layer. The dropout probability was set to 0.55 for both layers. The “binary cross-entropy” loss function was used. The “Adam” optimizer was used. The number of epochs was set to 80, and the batch size was set to 200.

2.2 Extended module

Hyperparameters of the 1D convolution layer: the length of the convolution layer was set to 8, the number of convolutional kernels was set to 600, and the "ReLU" activation function was used.
Hyperparameters of the full connection layer: the number of nodes was set to 600, and the "ReLU" activation function was used.
Other hyperparameters: In the training process, two dropout layers were deposited between the batch normalization layer and the full connection layer and between the full connection layer and the softmax layer. The dropout probability was set to 0.5 for both layers. The "categorical cross-entropy" loss function was used. The "Adam" optimizer was used. The number of epochs was set to 80, and the batch size was set to 256.

3 The performance of DeePVP with different hyperparameters on the PVP identification task

We tested the performance of DeePVP with different numbers of convolutional kernels and different kernel lengths on the PVP identification task. When training DeePVP using different numbers of convolutional kernels, the kernel length remained the same as that of the original CNN, and when training the DeePVP using different kernel lengths, the number of kernels remained the same as that of the original CNN. The results are shown in Figure S1 and Figure S2. The results show that the performance of DeePVP with other hyperparameters was not better than that of the CNN with the original hyperparameters, indicating that the hyperparameter design of DeePVP is reliable.

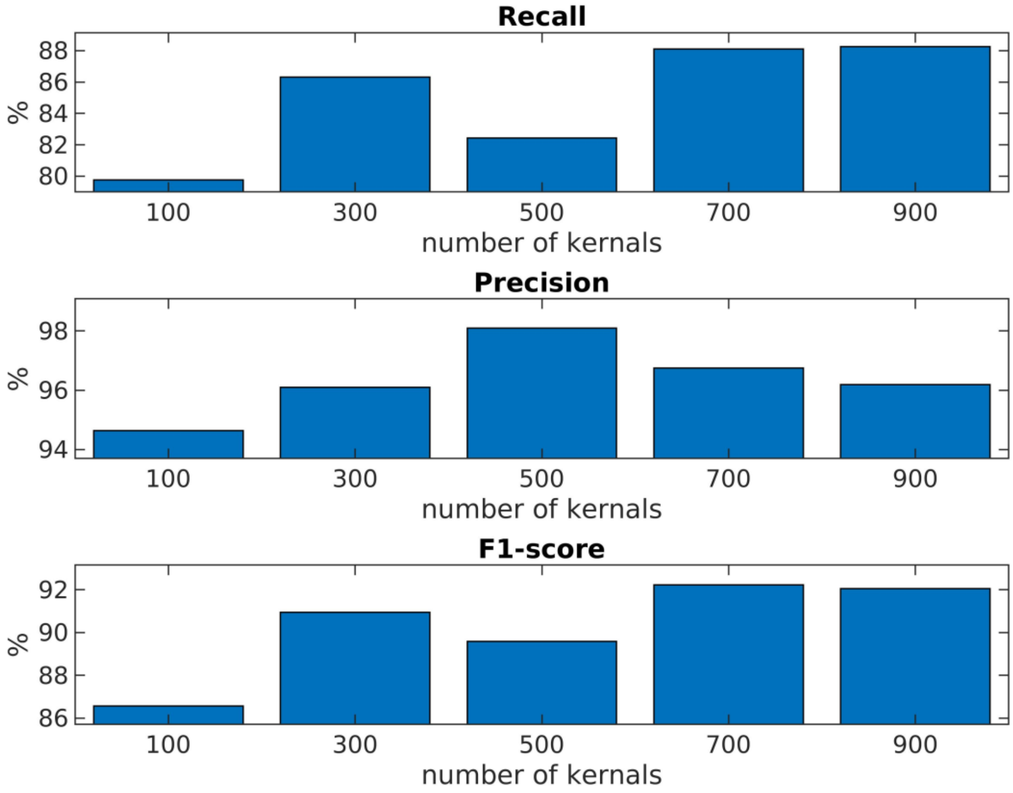

Figure S1. The performance of DeePVP with different numbers of convolutional kernels.

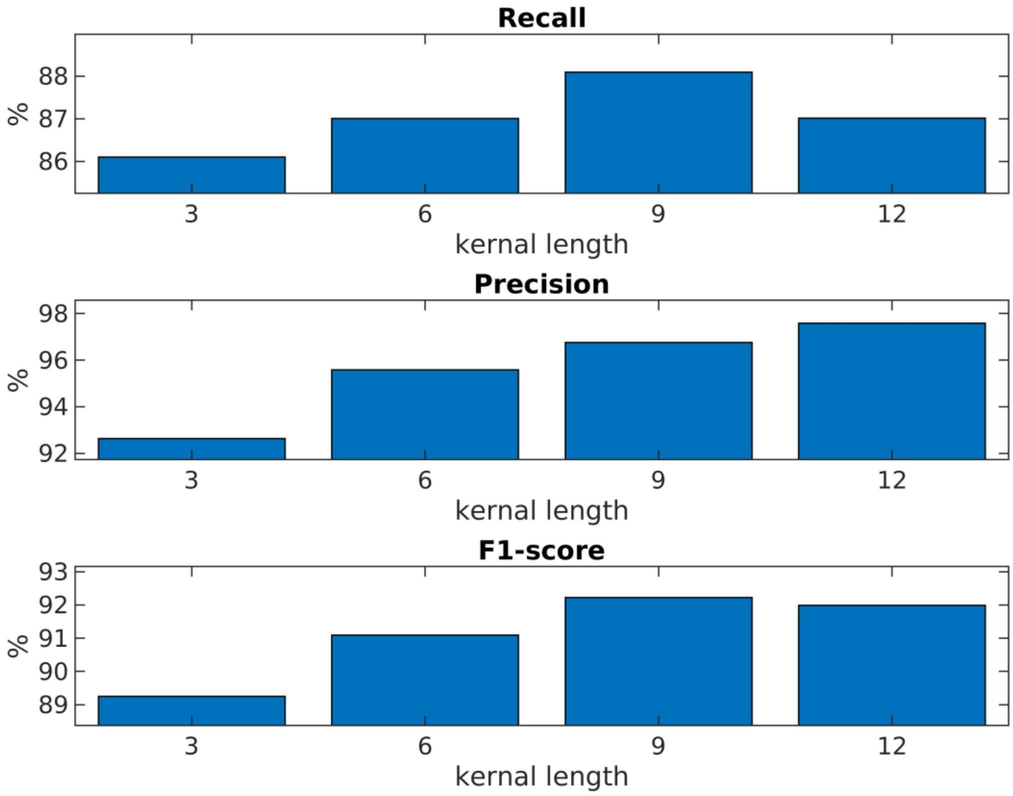

Figure S2. The performance of DeePVP with different kernel lengths.

4 The performance of DeePVP in the PVP identification task under different thresholds

We tested the *recall*, *precision* and *F1-score* of DeePVP in the PVP identification task under different thresholds. The results are shown in Figure S3.

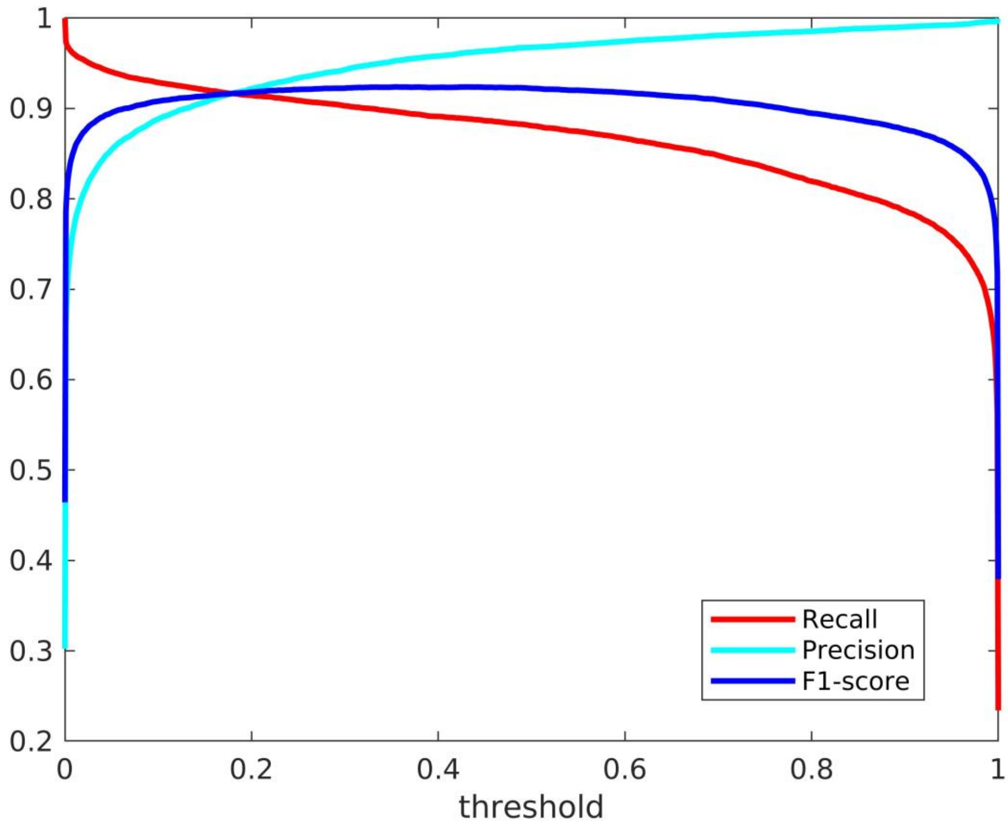

Figure S3. The performance of DeePVP in the PVP identification task under different thresholds.

5 The performance of 10-fold cross validation of DeePVP on the PVP classification task

Table S1. The average of *recall*, *precision*, *F1-score* and *accuracy* of the extended module of DeePVP in the 10-fold cross validation.

Category	*Recall* (%)	*Precision* (%)	*F1-score* (%)	*Accuracy* (%)
Major capsid	96.79	93.52	95.06	NA
Minor capsid	60.07	53.12	51.56	
Baseplate	77.28	88.56	82.10	
Major tail	87.63	80.77	83.93	
Minor tail	78.40	84.88	80.59	
Portal	91.40	93.02	92.13	
Tail fiber	65.72	65.87	64.54	
Tail sheath	90.38	95.51	92.41	
Collar	71.14	72.01	70.47	
Head-Tail Joining	91.32	88.92	89.85	
All		NA		**90.19**

6 PVP category predicted by PhANNs on the *Escherichia* phage phiEC1 genome

Table S2. PVP category predicted by PhANNs on the *Escherichia* phage phiEC1 genome.

Protein ID	PhANNs prediction
YP_009965634.1	HTJ
YP_009965637.1	Minor Capsid
YP_009965683.1	Portal
YP_009965687.1	Tail Fiber
YP_009965735.1	Minor Capsid
YP_009965770.1	Minor Capsid
YP_009965782.1	HTJ
YP_009965788.1	Portal
YP_009965791.1	Tail Fiber
YP_009965792.1	Portal
YP_009965794.1	Tail Fiber
YP_009965795.1	Baseplate
YP_009965796.1	Baseplate
YP_009965797.1	Collar
YP_009965798.1	Baseplate
YP_009965805.1	Tail Shaft
YP_009965806.1	Major Tail
YP_009965807.1	Portal
YP_009965808.1	HTJ
YP_009965809.1	Minor Capsid
YP_009965812.1	Major Capsid
YP_009965814.1	Portal
YP_009965818.1	Major Tail
YP_009965825.1	Baseplate
YP_009965826.1	Baseplate
YP_009965828.1	Tail Fiber
YP_009965830.1	Major Capsid
YP_009965831.1	Baseplate
YP_009965832.1	Tail Fiber
YP_009965834.1	Baseplate
YP_009965877.1	Tail Fiber
YP_009965878.1	Major Tail
YP_009965879.1	Tail Fiber
YP_009965880.1	Tail Fiber

Note: In the output file of PhANNs, “HTJ” refers to head-tail joining, and “Tail Shaft” refers to tail sheath.

7 DeePVP performance on partial genes

We tested the performance of DeePVP on partial genes in the PVP identification task (Figure S4) and PVP classification task (Figure S5). For each sequence in the testing data, 4 subsequences with 90%, 75%, 50% and 25% full length were randomly extracted.

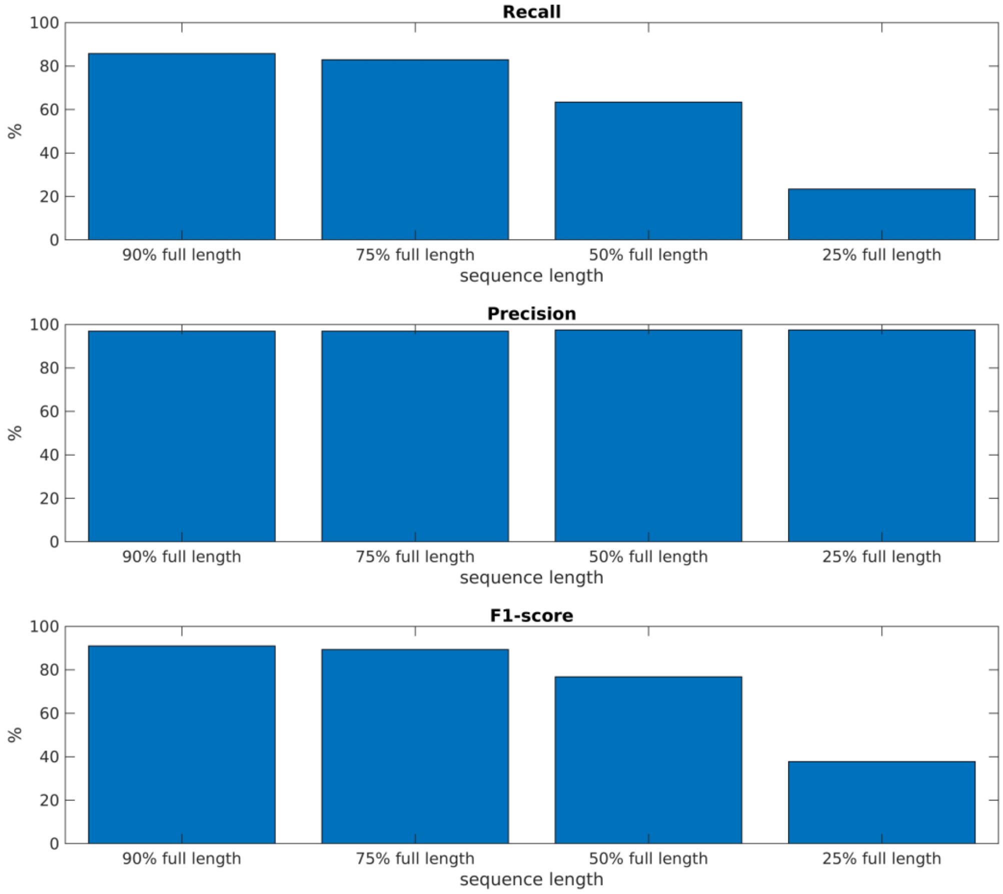

Figure S4. DeePVP performance on partial genes in the PVP identification task.

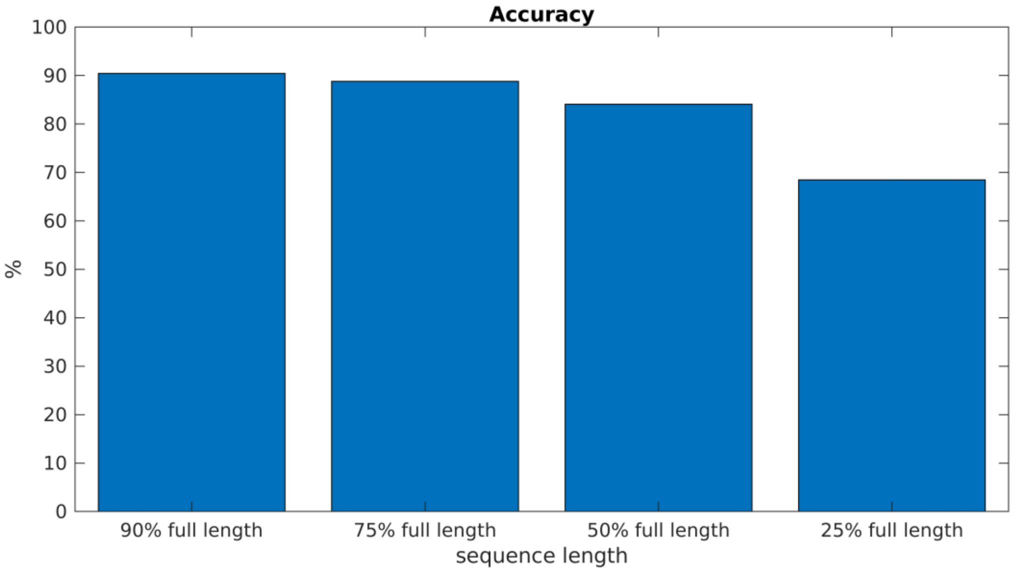

Figure S5. DeePVP performance on partial genes in the PVP classification task.
