# Supplementary material for: DeePVP: Identification and classification of phage virion proteins using deep learning
Source: Gigascience. 2022 Aug 11;11:giac076. doi: 10.1093/gigascience/giac076 (PMC9366990; doi:10.1093/gigascience/giac076)
Supplement: giac076_Response_to_Reviewer_Comments_Original_Submission [file giac076_response_to_reviewer_comments_original_submission.pdf]

Author's Response To Reviewer Comments

Close

We have uploaded all the responses in the cover letter as a supplementary material.

Close
