# Supplementary material for: DeePVP: Identification and classification of phage virion proteins using deep learning
Source: Gigascience. 2022 Aug 11;11:giac076. doi: 10.1093/gigascience/giac076 (PMC9366990; doi:10.1093/gigascience/giac076)
Supplement: giac076_Response_to_Reviewer_Comments_Revision_2 [file giac076_response_to_reviewer_comments_revision_2.pdf]

Author's Response To Reviewer Comments

Close

Dear editor, we have updated the REf #38 with the final GigaDB DOI citation, and uploaded a Word version (editable) of our manuscript for production.

Close
