# Supplementary material for: DeePVP: Identification and classification of phage virion proteins using deep learning
Source: Gigascience. 2022 Aug 11;11:giac076. doi: 10.1093/gigascience/giac076 (PMC9366990; doi:10.1093/gigascience/giac076)
Supplement: giac076_Reviewer_1_Report_Original_Submission — Satoshi Hiraoka -- 3/9/2022 Reviewed [file giac076_reviewer_1_report_original_submission.pdf]

Reviewer Report

Title: DeePVP: Identification and classification of phage virion proteins using deep learning

Version: Original Submission **Date:** 3/9/2022

Reviewer name: Satoshi Hiraoka

Reviewer Comments to Author:

In this manuscript, the authors developed a new tool, DeePVP, for predicting Phage Virion Proteins (PVPs) using the Deep learning approach. The purpose of this study is meaningful. As the authors described in the Introduction section, currently it is difficult to annotate functions of viral genes precisely because of its huge sequence diversity and existence of many unknown functions, and there are still many rooms to improve the performance of in silico annotation of phage genes including PVPs. Although I'm not an expert in machine learning, the newly proposed method based on Deep learning seems to be appropriate. The proposed tool showed clear outperformance compared with the other previously proposed tools, and thus, the tool might be valuable for further deep analysis of many viral genomes. Indeed, the authors conducted two case studies using real phage genomes and reported novel findings that may have insight into the genomics of the phages. Overall, the manuscript is well written, and I feel the tool has a good potential to contribute to the wide fields of viral genomics.
Unfortunately, I have concerns including the source cord openness. Also, I have some suggestions that would increase the clarity and impact of this manuscript if addressed.
Major:
I did not find DeePVP source cord on the GitHub page. Is the tool not open source? I strongly recommend the author disclose all scripts of the tool for further validation and secondary usage by other scientists. Or, at least, clearly state why the source cords need to hold private.
Also, I was much confused about the GitHub page because the uploaded files are not well structured. Scripts and data used for performance evaluation were included in 'data.zip' file, which should be renamed to be an appropriate one. 'Source code' button in the Releases page strangely links to the 'Supporting_data.zip' files which only contained installing manual but not source cord file. The authors should prepare the GitHub page appropriately that, for example, upload all source cords to the 'main' branch rather than include them in zip file, and 'source code' file in Releases should contain actual source code files rather than manual PDF.
According to the Material and method section, 1) using the Deep learning approach, and 2) using the large dataset retrieved from PhANNs as teacher dataset, are two of the important improvement from the other studies in the PVP identification task. Someone may suspect the better performance of DeePVP was mostly contributed by the increased teaching dataset rather than the used classification method. Is there a possibility that the previously proposed tools (especially the tools except for PhANNs) with re-training using the large PhANNs dataset could reach better performances than DeePVP?
The naming of 'Reliability index' (L249) is inaccurate. The score did not support the prediction 'reliability' (i.e., whether the predicted genes are truly PVP or not) but just reflects the fact that the gene is predicted as PVP by many tools without considering whether it is correct or incorrect. The sentence 'A

higher n indicates that this protein is predicted as PVP by more tools at the same time, and therefore, the prediction may be more reliable.' in L252 is not logical.
I dose not fully agree with the discussion that the tool will facilitate viral host prediction as mentioned in L294-302. It is very natural that if the phages are phylogenetically close and possess similar genomic structures including PVP-enriched regions, those will infect the same microbial lineage as a host. However, this is not evaluated systematically in wide phage lineages. In general, almost all phage-host relations are unknown in nature except few numbers of specific viruses such as E. Coli phages. Further detailed studies should be needed on whether and how degree the conservation of PVP-enriched region could be a potentially good feature to predict phage-host relationship. I think the phage-host prediction is beyond the scope of this tool, and thus the analysis could be deleted in this manuscript or just briefly mention in the Discussion section as a future perspective.
Minor:
The URL of the GitHub page is better to describe in the last of the Abstract or inside of the main text in addition to the 'Availability of supporting source code and requirements' section. This will make it easy for many readers to access the homepage and use the tool.
Fig 2 and 3. I think it is better to change the labels of the x-axis like 0 kb, 20 kb, 40 kb, ..., and 180 kb. This will make it easy for understanding that the horizontal bar represented the viral genome.

Level of Interest

Please indicate how interesting you found the manuscript: Choose an item.

Quality of Written English

Please indicate the quality of language in the manuscript: Choose an item.

Declaration of Competing Interests

Please complete a declaration of competing interests, considering the following questions:

- Have you in the past five years received reimbursements, fees, funding, or salary from an organisation that may in any way gain or lose financially from the publication of this manuscript, either now or in the future?
- Do you hold any stocks or shares in an organisation that may in any way gain or lose financially from the publication of this manuscript, either now or in the future?
- Do you hold or are you currently applying for any patents relating to the content of the manuscript?
- Have you received reimbursements, fees, funding, or salary from an organization that holds or has applied for patents relating to the content of the manuscript?
- Do you have any other financial competing interests?
- Do you have any non-financial competing interests in relation to this paper?

If you can answer no to all of the above, write 'I declare that I have no competing interests' below. If your reply is yes to any, please give details below.

I declare that I have no competing interests.

I agree to the open peer review policy of the journal. I understand that my name will be included on my report to the authors and, if the manuscript is accepted for publication, my named report including any attachments I upload will be posted on the website along with the authors' responses. I agree for my report to be made available under an Open Access Creative Commons CC-BY license (http://creativecommons.org/licenses/by/4.0/). I understand that any comments which I do not wish to be included in my named report can be included as confidential comments to the editors, which will not be published.

Choose an item.

To further support our reviewers, we have joined with Publons, where you can gain additional credit to further highlight your hard work (see: https://publons.com/journal/530/gigascience). On publication of this paper, your review will be automatically added to Publons, you can then choose whether or not to claim your Publons credit. I understand this statement.

Yes Choose an item.
