# Supplementary material for: DeePVP: Identification and classification of phage virion proteins using deep learning
Source: Gigascience. 2022 Aug 11;11:giac076. doi: 10.1093/gigascience/giac076 (PMC9366990; doi:10.1093/gigascience/giac076)
Supplement: giac076_Reviewer_2_Report_Original_Submission — Deyvid Amgarten -- 3/19/2022 Reviewed [file giac076_reviewer_2_report_original_submission.pdf]

Reviewer Report

Title: DeePVP: Identification and classification of phage virion proteins using deep learning

Version: Original Submission **Date:** 3/19/2022

Reviewer name: Deyvid Amgarten

Reviewer Comments to Author:

The manuscript presents DeePVP, a new tool for PVP annotation of a phage genome. The tool implements two separate modules: The main module aims to discriminate PVPs from non-PVPs within a phage genome, while the extended module of DeePVP can further classify predicted PVPs into the ten major classes of PVPs. Compared with the present state-of-the-art tools, the main module of DeePVP performs better, with a 9.05% higher F1-score in the PVP identification task. Moreover, the overall accuracy of the extended module of DeePVP in the PVP classification task is approximately 3.72% higher than that of PhANNs, a known tool in the area.

Overall, the manuscript is well written, clear, and I could not identify any serious methodological inconsistence.

I was not sure whether to consider the performance metrics shown as significant improvements or not, since PhANNs already does a similar job on that regard. And it is better for some types of PVPs for example. But I would rather give this task to readers and other researchers in the area. Specifically, I enjoyed the discussion about how one-hot encoded features may be more suitable for predictions that k-mers based ones. And by consequence, that convolution networks may present an advantage against simple multilayer perceptron networks. This manuscript brings an important contribution to the phage genomics and machine learning fields. I am certain that DeePVP will be helpful to many researchers.

I have a major question about the composition of the dataset used to train the main module: Among the PVP proteins, do authors know if only the ten types of PVP are present? There is a rapid mention to key words used to assemble the PhANNs dataset in the discussion (line 340), but that is not clear to me. This will help me understand the following:

Line 124: The CNN in the extended module has an output softmax layer, which outputs likelihood scores for 10 types of virion proteins. I wonder if only proteins from these 10 types were included in the datasets used to train the CNNs. I mean, is it possible that a different type of virion protein is predicted by the main module as PVP? And if so, how would the extended module predict this protein since it is PVP but none of the ten types?

Minors:

Line 121: By default, a protein with a PVP score higher than 0.5 is regarded as a PVP. How was this cutoff chosen? Was this part of the k-cross validation process?

Line 157 and other pieces in the manuscript: I would suggest authors not to use sentences like "F1-score is 9.05% much higher than that of PhANNs" for obvious reasons that 9% may not seem such a great difference for using the "much" adverb. Same thing to "much better" and variations.

About the comparisons between DeePVP and PhANNs: Did authors make sure that instances of the test set were not used to train the PhANNs model being used?

Line 221: What authors mean by "more authentic prediction"?
Looking at the github repository, I found rather unusual that authors chose to upload only a PDF with instructions of how to use and install. It is very detailed, I appreciate. The virtual machine and docker containers are also nice resources to help less experienced users. However, I noticed that the github repository has no clear mention to the source code of the tool. I found it by a mention in the Availability of supporting data, where authors created a release with the datasets and the scripts. Again, very unusual, but I suppose authors have chosen this approach due to github limitations to large files.
Table 2: I would like to ask authors what might me the reason for such low performance metrics to some types of PVP (for example, minor capsid)?
Figure 5 states: "Host genus composition of the subject sequences". But there is a "Myoviridae" category, which is a family of phages. Not anything related to bacterial hosts. Please, verify why this is in the figure.

Level of Interest

Please indicate how interesting you found the manuscript: Choose an item.

Quality of Written English

Please indicate the quality of language in the manuscript: Choose an item.

Declaration of Competing Interests

Please complete a declaration of competing interests, considering the following questions:

- Have you in the past five years received reimbursements, fees, funding, or salary from an organisation that may in any way gain or lose financially from the publication of this manuscript, either now or in the future?
- Do you hold any stocks or shares in an organisation that may in any way gain or lose financially from the publication of this manuscript, either now or in the future?
- Do you hold or are you currently applying for any patents relating to the content of the manuscript?
- Have you received reimbursements, fees, funding, or salary from an organization that holds or has applied for patents relating to the content of the manuscript?
- Do you have any other financial competing interests?
- Do you have any non-financial competing interests in relation to this paper?

If you can answer no to all of the above, write 'I declare that I have no competing interests' below. If your reply is yes to any, please give details below.

I declare that I have no competing interests

I agree to the open peer review policy of the journal. I understand that my name will be included on my report to the authors and, if the manuscript is accepted for publication, my named report including any attachments I upload will be posted on the website along with the authors' responses. I agree for my report to be made available under an Open Access Creative Commons CC-BY license (http://creativecommons.org/licenses/by/4.0/). I understand that any comments which I do not wish to be included in my named report can be included as confidential comments to the editors, which will not be published.

Choose an item.

To further support our reviewers, we have joined with Publons, where you can gain additional credit to further highlight your hard work (see: https://publons.com/journal/530/gigascience). On publication of this paper, your review will be automatically added to Publons, you can then choose whether or not to claim your Publons credit. I understand this statement.

Yes Choose an item.
