# Supplementary material for: DeePVP: Identification and classification of phage virion proteins using deep learning
Source: Gigascience. 2022 Aug 11;11:giac076. doi: 10.1093/gigascience/giac076 (PMC9366990; doi:10.1093/gigascience/giac076)
Supplement: giac076_Reviewer_2_Report_Revision_1 — Deyvid Amgarten -- 6/16/2022 Reviewed [file giac076_reviewer_2_report_revision_1.pdf]

Reviewer Report

Title: DeePVP: Identification and classification of phage virion proteins using deep learning

Version: Revision 1 **Date:** 6/16/2022

Reviewer name: Deyvid Amgarten

Reviewer Comments to Author:

Thank you for authors' responses. Most of my concern were addresses.
I have to say, though, that the github page is not quite in the standards for a bioinformatics tools yet. I appreciate the source code upload, but I noticed that not a single line of #comments were present in the code I have checked. README file is also not very clarifying.
I do not consider this as an impediment for publication (since there are detailed info in GigaScience DB), but perhaps this may hind usage of authors' tool. Most users will only look at the github repository.
I suggest some improvements in case authors judge my comment makes some sense. Bellow I list three examples just to give authors an idea:
https://github.com/fenderglass/Flye
https://github.com/LaboratorioBioinformatica/MARVEL
https://github.com/vrmarcelino/CCMetagen
One last concern was about authors' response to the Myoviridae mistake in figure 5. Authors stated that the genus of a phage host is in its name (as for example Escherichia phage XX). But this is a dangerous assumption, since many phage names are outside of this rule. For example, there are many phages with Enterobacteria phage XXX (for instance NC_054905.1), meaning that they infect some Enterobacteria. Again, enterobacteria is not a genus. Phage nomenclature may be a mess sometimes, be careful.

Level of Interest

Please indicate how interesting you found the manuscript: Choose an item.

Quality of Written English

Please indicate the quality of language in the manuscript: Choose an item.

Declaration of Competing Interests

Please complete a declaration of competing interests, considering the following questions:

- Have you in the past five years received reimbursements, fees, funding, or salary from an organisation that may in any way gain or lose financially from the publication of this manuscript, either now or in the future?
- Do you hold any stocks or shares in an organisation that may in any way gain or lose financially from the publication of this manuscript, either now or in the future?

- Do you hold or are you currently applying for any patents relating to the content of the manuscript?
- Have you received reimbursements, fees, funding, or salary from an organization that holds or has applied for patents relating to the content of the manuscript?
- Do you have any other financial competing interests?
- Do you have any non-financial competing interests in relation to this paper?

If you can answer no to all of the above, write 'I declare that I have no competing interests' below. If your reply is yes to any, please give details below.

I declare that I have no competing interests

I agree to the open peer review policy of the journal. I understand that my name will be included on my report to the authors and, if the manuscript is accepted for publication, my named report including any attachments I upload will be posted on the website along with the authors' responses. I agree for my report to be made available under an Open Access Creative Commons CC-BY license (http://creativecommons.org/licenses/by/4.0/). I understand that any comments which I do not wish to be included in my named report can be included as confidential comments to the editors, which will not be published.

Choose an item.

To further support our reviewers, we have joined with Publons, where you can gain additional credit to further highlight your hard work (see: https://publons.com/journal/530/gigascience). On publication of this paper, your review will be automatically added to Publons, you can then choose whether or not to claim your Publons credit. I understand this statement.

Yes Choose an item.
